# Sesquiterpene lactones as emerging biomolecules to cease cancer by targeting apoptosis

**DOI:** 10.3389/fphar.2024.1371002

**Published:** 2024-03-11

**Authors:** Chou-Yi Hsu, Sadegh Rajabi, Maryam Hamzeloo-Moghadam, Abhinav Kumar, Marc Maresca, Pallavi Ghildiyal

**Affiliations:** ^1^ Department of Pharmacy, Chia Nan University of Pharmacy and Science, Tainan, Taiwan; ^2^ Traditional Medicine and Materia Medica Research Center, Shahid Beheshti University of Medical Sciences, Tehran, Iran; ^3^ Traditional Medicine and Materia Medica Research Center and Department of Traditional Pharmacy, School of Traditional Medicine, Shahid Beheshti University of Medical Sciences, Tehran, Iran; ^4^ Department of Nuclear and Renewable Energy, Ural Federal University Named After the First President of Russia Boris Yeltsin, Ekaterinburg, Russia; ^5^ Aix Marseille Univ, CNRS, Centrale Marseille, iSm2, Marseille, France; ^6^ Uttaranchal Institute of Pharmaceutical Sciences, Uttaranchal University, Dehradun, India

**Keywords:** cancer, sesquiterpene lactones, apoptosis, evasion, intrinsic, extrinsic

## Abstract

Apoptosis is a programmed cell death comprising two signaling cascades including the intrinsic and extrinsic pathways. This process has been shown to be involved in the therapy response of different cancer types, making it an effective target for treating cancer. Cancer has been considered a challenging issue in global health. Cancer cells possess six biological characteristics during their developmental process known as cancer hallmarks. Hallmarks of cancer include continuous growth signals, unlimited proliferation, resistance to proliferation inhibitors, apoptosis escaping, active angiogenesis, and metastasis. Sesquiterpene lactones are one of the large and diverse groups of planet-derived phytochemicals that can be used as sources for a variety of drugs. Some sesquiterpene lactones possess many biological activities such as anti-inflammatory, anti-viral, anti-microbial, anti-malarial, anticancer, anti-diabetic, and analgesic. This review article briefly overviews the intrinsic and extrinsic pathways of apoptosis and the interactions between the modulators of both pathways. Also, the present review summarizes the potential effects of sesquiterpene lactones on different modulators of the intrinsic and extrinsic pathways of apoptosis in a variety of cancer cell lines and animal models. The main purpose of the present review is to give a clear picture of the current knowledge about the pro-apoptotic effects of sesquiterpene lactones on various cancers to provide future direction in cancer therapeutics.

## 1 Introduction

As a world health challenge, cancer has affected more than 17 million people in 2018 and over 8 million of these patients died from the disease. Moreover, recent estimates reveal that number of new cases can increase to more than 27 million by 2040 (Rajabi et al., 2021). Cancer therapy is a complicated process. Conventional cancer therapies include surgery, chemotherapy, and radiotherapy, while a number of novel methods such as targeted therapeutic strategies affecting molecular pathways involved in cancer development, promotion, and progression have been improved dramatically (Debela et al., 2021). Each tumor obtains six biological characteristics during its developmental process known as cancer hallmarks. Hallmarks of cancer include continuous growth signals, unlimited proliferation, resistance to proliferation inhibitors, apoptosis escaping, active angiogenesis, and metastasis (Hanahan, 2022). Targeting each of these processes can be considered an efficient therapeutic strategy to halt cancer.

Apoptosis is a programmed cell death mechanism with a physiological role to eliminate a single cell from the living tissue (Bedoui et al., 2020). Apoptosis, as a physiological mechanism, controls the unlimited expansion of the cell population either to keep tissue homeostasis or to eliminate harmful cells with vast DNA damage (Morana et al., 2022). Any pathological transformation that occurs in the tissue may trigger dysregulation of this physiological process leading to the development of some diseases, especially cancer (Koff et al., 2015). The induction of apoptosis has been reported as an effective approach to halt cancer cell growth cancer (Carneiro and El-Deiry, 2020). Phytochemical compounds, as secondary plant metabolites, have been shown as promising candidates for cancer therapy by inducing apoptotic cell death in cancer cells (Wani et al., 2023).

Sesquiterpene lactones are one of the large and diverse groups of planet-derived phytochemicals with a 15-carbon backbone structure (Matos et al., 2021). These phytochemicals have various biological activities such as anti-inflammatory, anti-viral, anti-microbial, anti-malarial, anticancer, anti-diabetic, and analgesic (Sokovic et al., 2017). Sesquiterpene lactones are derivatives of isopentenyl diphosphate and dimethylallyl diphosphate, which are the mediators of mevalonate and 2-C-methyl-D-erythritol pathways in the cytosol and chloroplast, respectively (Matos et al., 2021). Therefore, the main purposes of this review are to summarize apoptosis mechanisms, dysregulation of apoptosis during cancer development, and sesquiterpene lactones with the ability to target apoptosis in cancer cells.

## 2 Mechanisms of apoptotic cell death

Apoptosis has been shown as a stage-dependent process. During the early stages of apoptosis, condensation of chromatin and nuclear takes place (Lossi, 2022). In late apoptosis, major events include cell shrinkage, plasma membrane blebbing, modification of organelles, loss of cell membrane integrity, and formation of apoptotic bodies (Green, 2022). The exposure of phosphatidylserine (PS) molecules on the outer layer of the cell membrane is a signal, known as “Eat me,” to attract phagocytes to engulf apoptotic cells (Segawa and Nagata, 2015). Apoptosis is mainly triggered in two different ways, including extrinsic or intrinsic pathways, which are described in the next section.

### 2.1 Extrinsic pathway

The extrinsic pathway of apoptosis is triggered from extracellular space by a direct connection between specific extracellular molecules, known as death ligands, and cell surface proteins, which are named death receptors (Lossi, 2022). To activate the intracellular cascade of the extrinsic pathway of apoptosis, death ligands, including Fas, TNF-related apoptosis-inducing ligand (TRAIL), and tumor necrosis factor (TNF) directly bind Fas receptor (FasR), TNF receptors, and TRAIL receptors on the cell surface (Green, 2022). Death receptors contain an intracellular conserved protein-protein interaction domain known as the death domain, which is binding sites for adaptor proteins, such as TNF receptor-associated death domain (TRADD) and Fas-associated death domain (FADD) as well as pro-caspase 8 and 10 (Huang et al., 1996; Cao et al., 2011; Lee et al., 2012b). An intracellular FADD like IL-1β -converting enzyme (FLICE) inhibitory protein (c-FLIP) blocks the first step of the extrinsic pathway by inhibiting procaspases (Humphreys et al., 2018). This anti-apoptotic protein has three different isoforms including c-FLIP(L), c-FLIP(S), and c-FLIP(R), which have anti-apoptosis activity during both physiologic and pathologic conditions by acting on caspase-8/10 activity (Bagnoli et al., 2010). The other inhibitors of the extrinsic pathway are decoy receptors (DcRs), which are cell-surface proteins with a role as competitive inhibitors of death receptors in binding to death ligands. DcR1 and DcR2 have been shown to bind TRAIL receptors and DcR3 acts as a decoy receptor for FasL in several cancer cells (Soto-Gamez et al., 2022). When activated, caspases 8 and 10 directly cleave effector caspases (3, 6, and 7) to activate them. They also cleave BH3 interacting domain death agonist (BID), which migrates into the mitochondria to trigger the intrinsic pathway of apoptosis by releasing cytochrome C into the cytoplasm (Huang et al., 2016). Thus, the intrinsic pathway of apoptosis can be initiated by indirect action of the extrinsic cascade.

### 2.2 Intrinsic pathway

**FIGURE 1:**
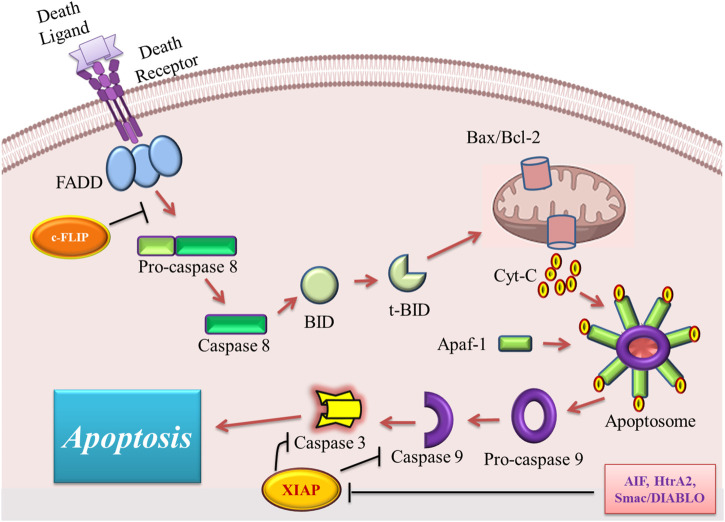
An overview of the extrinsic and intrinsic pathways of apoptosis and their major modulators. FADD, Fas-associated death domain; Bax, Bcl2 associated X; Bcl-2, B cell lymphoma-2; Cyt C, cytochrome C; Apaf-1, apoptotic protease activating factor 1; AIF, apoptosis-inducing factor; Smac/DIABLO, second mitochondria-derived activator of caspase/direct IAP binding protein with low pI; HtrA2, Omi/high-temperature requirement protein A; XIAP, X-linked inhibitor of apoptosis protein; c-FLIP, cellular FADD-like IL-1β-converting enzyme (FLICE) inhibitory protein; t-BID, truncated BH3 interacting-domain death agonist.

The stimulation of the intrinsic pathway of apoptosis is under the control of some intracellular inducers (D'Arcy, 2019). These stimulators include oxidative stress, irradiation, cytotoxic drugs, DNA damage, and hypoxia (Mehrzadi et al., 2021). The intrinsic pathway is known as the mitochondrial pathway because it is mediated by mitochondria in the intracellular space (D'Arcy, 2019). The initial step in the induction of the intrinsic pathway is the enhancement of the permeability of the mitochondrial outer membrane, which leads to the release of cytochrome C into the cytoplasm of the cell (Zhang et al., 2017). Cytochrome C forms a multiprotein complex structure, known as the apoptosome, with apoptotic protease activating factor-1 (Apaf-1) and procaspase-9 in the cytosol (Zhang et al., 2017). Different factors are involved in the stimulation of cytochrome C release from mitochondria to cytosol including increased mitochondrial permeability transition (MPT), elevated ratio of proapoptotic B-cell lymphoma protein 2 (Bcl-2) family proteins, and hypotonicity of cytoplasm due to ionic effluxes (Naumova and Šachl, 2020). Among these factors, the Bcl-2 family members are the major regulators of the intrinsic pathway. This family contains two sets of proteins with two opposite roles in the induction of the intrinsic pathway of apoptosis. For example, anti-apoptotic proteins of this family including Bcl-2, Bcl-xL, Bcl-W, Bfl-1, and Mcl-1 inhibit apoptosis induction, but pro-apoptotic ones Bax, Bak, Bad, Bcl-Xs, Bid, Bik, Bim, and Hrk promote the initiation of apoptosis (Opferman and Kothari, 2018). The main function of the proapoptotic members of the Bcl-2 family is to perforate the mitochondrial membrane to help cytochrome C efflux; however anti-apoptotic members act by inhibiting the function of proapoptotic proteins (Uren et al., 2017). Upon the release of cytochrome C, three proteins also enter the cytosol. These proteins are apoptosis-inducing factor (AIF), the second mitochondria-derived activator of caspase (Smac)/direct IAP binding protein with low pI (DIABLO), and Omi/high-temperature requirement protein A (HtrA2), which bind to the inhibitor of apoptosis proteins (IAPs) and block these proteins. In the absence of the above-mentioned proteins, IAPs bind and inhibit caspase-9 and -3 leading to the inhibition of apoptosis (Flores-Romero et al., 2020). Caspase-9 is generally in its inactive form, called procaspase-9, which is activated by the function of apoptosome leading to the activation of effector caspases and cell destruction (Bratton and Salvesen, 2010). Figure 1 illustrates the overview of the intrinsic and extrinsic pathways of apoptosis and their different modulators.

## 3 Apoptosis dysregulation in cancer

**FIGURE 2:**
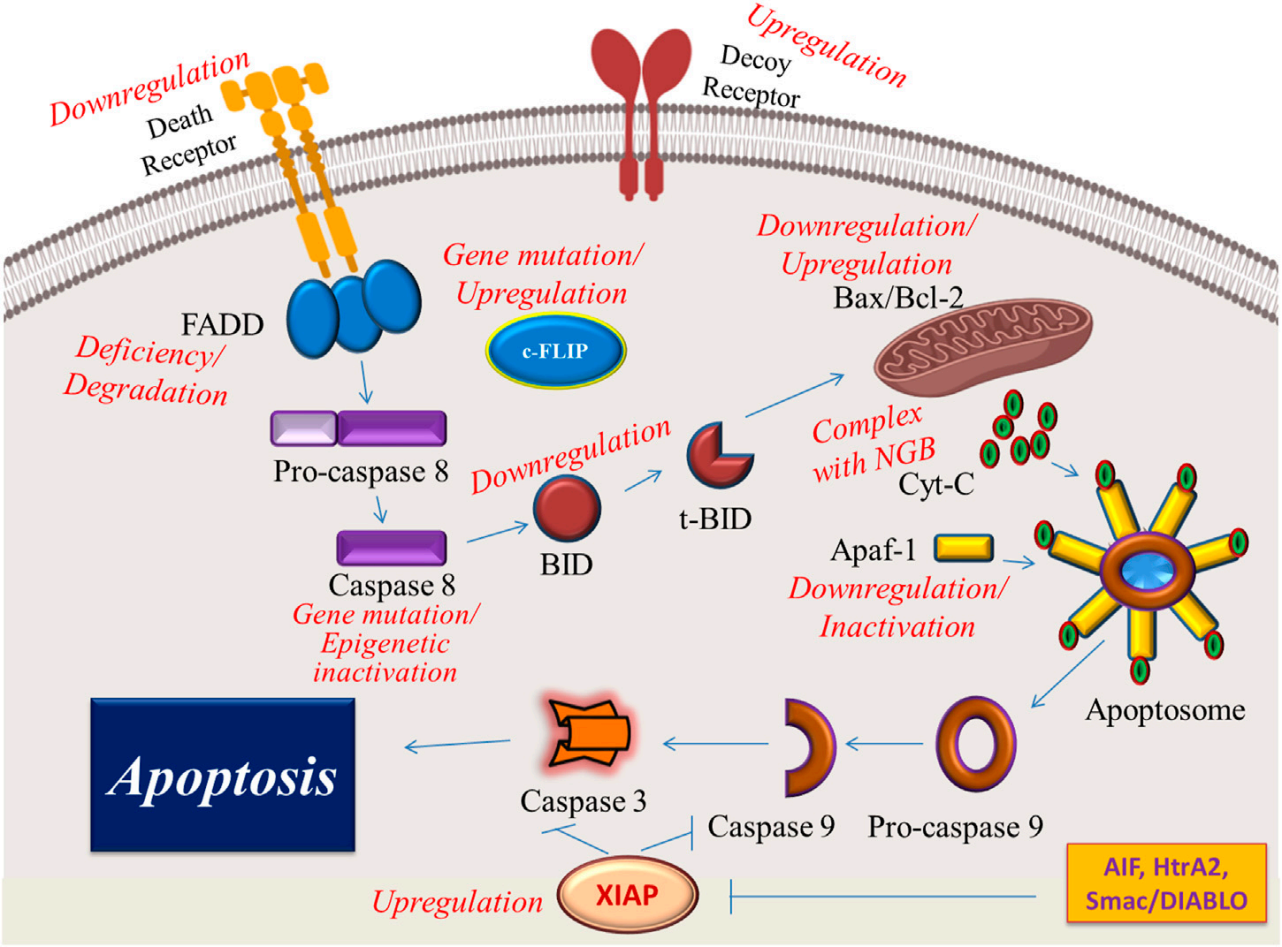
Illustrative presentation of the different mechanisms by which cancer cells can escape apoptosis. NGB, Neuroglobin is an oxygen-binding globin with overexpressed levels in some cancer cells that forms a complex with cytochrome C and prohibits its release into the cytoplasm.

Dysregulation of both intrinsic and extrinsic pathways of apoptosis leads to apoptosis evasion and therapy resistance in cancerous tumors (Neophytou et al., 2021). Therefore, new therapeutic approaches are committed to preventing cancer cells from evading apoptotic cell death as one of the hallmarks of cancer (Neophytou et al., 2021). These mechanisms of apoptosis evasion are summarized in Figure 2.

In the extrinsic pathway, death receptors are first-line molecules that may be affected in cancer cells. For example, therapy resistance in leukemia and neuroblastoma cells is attributed to CD95 downregulation on the cell surface of these cancer cells (Friesen et al., 1997). Downregulation of these receptors and lack of enough molecules on the cell surface of cancer cells may arise from some epigenetic alterations induced by cancerous conditions to evade apoptosis during cancer development (Manoochehri et al., 2016). Other cell surface receptors that may be used by cancer cells to escape apoptosis are DcRs. Glioblastoma, colon, and lung cancers amplify DcR3 to evade apoptosis by inhibiting Fas ligand-mediated cell death (Pitti et al., 1998). Death receptor-linked and soluble proteins in the cytosolic cascade of the extrinsic pathway are usually affected in cancer cells. Any dysregulation in the levels of FADD and cFLIP in renal carcinoma can hinder death receptor-mediated apoptosis and increase cell survival in this cancer (Ranjan et al., 2012). Loss of the expression or degradation of the FADD leads to apoptosis resistance in triple-negative breast cancer cells (Lee et al., 2012a). According to recent reports, 10% of human head and neck squamous cell carcinomas (HNSCCs) have a mutated caspase 8, which is responsible for the apoptosis-resistant phenotype in these patients (Li et al., 2014; Cui et al., 2021). In addition to genetic mutations, epigenetic mechanisms are also involved in the inactivation of caspase 8. For example, DNA methylation in the gene encoding caspase 8 results in the downregulation of this enzyme and leads to apoptosis escape in human malignant glioma cells (Teng et al., 2017).

Cancer cells also make some changes in factors involved in the intrinsic pathway to provide favorable conditions for apoptosis evasion. For example, some cancer cells upregulate Bcl-2 family proteins and downregulate proapoptotic protein Bax to prevent apoptotic cell death (Lopez et al., 2022). Downregulation of Bid in MCF-7 cells is associated with inhibition of TRAIL-induced caspase-8 activation (Sivaprasad et al., 2007). It has been reported that c-FLIP variants may cause resistance to death receptor ligands and chemotherapeutics in cancer cells. Furthermore, overexpression of c-FLIP is correlated with poor clinical results (Safa, 2013). Neuroglobin (NGB) is an oxygen-binding globin with overexpressed levels in some cancer cells (Fiocchetti et al., 2019). This protein has the ability to form a complex with cytochrome C, leading to the inhibition of cytochrome C release and blockage of apoptosis (Tiwari et al., 2018). It has been reported that downregulation and inactivation of Apaf-1 in skin and ovarian cancer cells resulted in apoptosis resistance and therapy failure (Tan et al., 2011; Tian et al., 2018). Overexpression of the X-linked inhibitor of apoptosis protein (XIAP) in many tumor cells has been reported as an adverse alteration that renders the cancer cell apoptosis-resistance capacity (Obexer and Ausserlechner, 2014).

## 4 Sesquiterpene lactones with pro-apoptotic activities in cancer

### 4.1 Alantolactone

A study on the apoptosis-inducing activity of alantolactone, isolated from *Inula helenium*, in MDA-MB-231 breast cancer cells showed that this agent stimulates mitochondrial-mediated apoptosis in these cells. Alantolactone increased the Bax/Bcl-2 ratio and cytochrome C release from mitochondria to the cytoplasm. It also decreased mitochondrial membrane potential (MMP) and activated caspase 9 and 3. Furthermore, alantolactone exerted its anticancer effect on MDA-MB-231 breast cancer cells through reactive oxygen species (ROS)-mediated mitochondrial dysfunction (Cui L. et al., 2018). In another study, alantolactone induced apoptosis of MCF-7 cells by downregulating Bcl-2, upregulating Bax and p53, and activating caspases 3 and 12 (Liu et al., 2018). In chronic myelogenous leukemia (CML) cell line, K562, alantolactone treatment resulted in apoptosis induction by activating caspase-3 and poly (ADP-ribose) polymerase-1 (PARP-1) as well as the reduction of MMP in these cancer cells. Alantolactone triggered apoptotic cell death in the K562 cell line by inhibiting the nuclear factor kappa B (NF-κB) pathway and tumor necrosis factor α (TNFα) function on this pathway (Wei et al., 2013). Treatment of the human colon cancer cell line, RKO, with alantolactone decreased proliferation and increased apoptosis in these cancer cells. Alantolactone treatment also led to the accumulation of intracellular ROS and the disruption of MMP in RKO cells. This phytochemical induced apoptosis in RKO cells by modulating mitochondria pathway via diminishing Bcl-2 expression and augmenting Bax protein levels with activating effects on caspase-3 and -9 (Zhang et al., 2013). Ren et al. (2021) conducted a study to evaluate the effect of alantolactone on colorectal cancer and its underlying mechanism. Alantolactone could induce HCT-116 cell apoptosis by downregulating Bcl-2, upregulating Bax, activation of caspase 3, MMP loss, and G0/G1 phase arrest. The xenograft tumor model of the colon cancer cells in Balb/c mice showed that Alantolactone at a dose of 50 mg/kg significantly reduced the tumor size. Treatment of gastric cancer cell lines BGC-823 and SGC-7901 with different concentrations of alantolactone induced apoptosis of these cells by increasing the expression of Bax and p53 and decreasing the expression of anti-apoptotic protein Bcl-2. These effects may arise from the modulation of mitogen-activated protein kinase (MAPK) and nuclear factor-κB (NF-κB) pathways (He et al., 2019). The most frequent type of malignant and aggressive brain tumor in adults is glioblastoma multiform (GBM). Khan et al. (2012b) demonstrated that alantolactone suppresses the growth of GBM cell line U87 by inducing apoptosis in these cancer cells. They reported that alantolactone triggered apoptosis of U87 cells through upregulation of p53 and Bax, downregulation of Bcl-2, cytochrome C release, and activation of caspases 9 and 3. In the hepatocellular carcinoma cell line, HepG2, alantolactone induced apoptosis in a dose-dependent manner. The apoptosis-inducing activity of alantolactone in HepG2 cells was shown to be associated with an increased ratio of Bax to Bcl-2 proteins and activation of caspase 3. This phytochemical also hampered MMP and led to the formation of ROS in these cancer cells (Khan et al., 2013). In another study on HepG2 cells, this sesquiterpene lactone triggered apoptosis by acting on both intrinsic and extrinsic pathways. It downregulated protein levels of Bcl-2, increased cytosolic cytochrome C, decreased MMP, and activated caspase 3, 9, and 8 leading to the cleavage and activation of Bid (Lei et al., 2012). In A549 lung adenocarcinoma cells, alantolactone induced apoptosis and enhanced the chemosensitivity of A549 cells to doxorubicin by inhibiting the JAK/STAT3 pathway in these cells. It exerted apoptosis-promoting effects via increasing the expression levels of Bax and Bad, activation of caspase 3 and 9 as well as a decrease in the levels of Bcl-2, survivin, and XIAP (Maryam et al., 2017). Alantolactone effects on the proliferation and apoptosis of human melanoma cells (A375 and B16) have been illustrated by upregulation of Bax expression, activation of caspase 3, and downregulation of Bcl-2 and the proliferation marker PCNA (Zhang L. et al., 2023). This sesquiterpene has been shown to stimulate apoptosis in human multiple myeloma cell lines (OPM2 and IM-9). Alantolactone induced apoptosis in these cell lines by activating caspase 3 and caspase 9 with no significant effect on caspase 8. This phytochemical also downregulated Bcl-2 and survivin and upregulated Bax in both cell lines. These data suggest that alantolactone specifically triggers the intrinsic pathway of apoptosis in multiple myeloma cells (Yao et al., 2015). Treatment of human oral squamous cell carcinoma (OSCC) cells including CAL27 and SCC9 with alantolactone had anti-proliferative and pro-apoptotic activity. The authors of that study have declared that alantolactone led to CAL27 and SCC9 cell injury by the formation of ROS, loss of MMP, and ATP depletion. However, they did not check the other prominent markers of apoptosis (Zhang Y. et al., 2023). Alantolactone treatment in 143B and MG63 osteosarcoma cell lines promoted apoptosis and arrested the cell cycle at the G2/M phase. Mechanistically, alantolactone declined the protein level of anti-apoptotic factor Bcl-2 while increasing the protein level of pro-apoptotic factors, Bax and Bad. Furthermore, it induced the activation of caspase 3 and PARP. Moreover, it decreased tumor growth and metastasis of 143B cells in a xenograft model of osteosarcoma (Yang C. et al., 2022). Alipour et al. (2022) examined the effect of cisplatin, alantolactone, and ZnO nanoparticles (ZnONPs) on the induction of apoptosis in the SKOV3 ovarian cancer cell line. Their data revealed that both alantolactone and ZnONPs increase the sensitivity of SKOV3 cells to cisplatin. They further showed that the cells treated with a single, double, or triple combination of the three agents had a significant downregulation in the expression of Bcl-2, XIAP, and cyclin D1 and remarkable upregulation of pro-apoptotic factor Bax. ROS production was also higher in the treatment groups than in the untreated control cells. Alantolactone promoted apoptosis and induced cell cycle G1/G0 phase arrest in squamous lung cancer SK-MES-1 cells. I upregulated the levels of caspases 3, 8, and 9 as well as the PARP enzyme and pro-apoptotic factor Bax. Bcl-2 was the only factor that was found to be downregulated in alantolactone-treated SK-MES-1 cells (Zhao et al., 2015). Another sesquiterpene lactone, called 13-O-acetylsolstitialin A (13ASA), which was isolated from *Centaurea cyanus*, has been shown to suppress the proliferation of MCF-7 and MDA-MB-231 breast cancer cell lines. Further experiments revealed that 13ASA stimulated cell cycle arrest at subG1 and G1 phases possibly by suppressing the cyclin D1 and Cdk-4 protein levels. 13ASA significantly upregulated Bax and downregulated Bcl-2 proteins both in MCF-7 and MDA-MB-231 cell lines. The mechanisms of apoptosis-inducing activity of this agent were reported to act by decreasing MMP and increasing ROS concentration in the intracellular spaces (Keyvanloo Shahrestanaki et al., 2019). Table 1 summarizes pro-apoptotic sesquiterpene lactones and their underlying mechanisms of action in diffident cancers.

**TABLE 1:** Sesquiterpene lactones with the ability to induce apoptosis in different cancers.

Molecule	Plant source	Concentration (µM)	Cancer type	Cancer model	Altered factors	References
Alantolactone	*Inula helenium*	5, 10, and 15 μM	Breast	MDA-MB-231 cell line	Induced: Bax, cytochrome C release, activation of caspase 3 and 9	Cui et al. (2018a)
					Reduced: Bcl-2, MMP	
		10, 20, and 30 μM	Breast	MCF-7 cell line	Induced: Bax, activation of caspase 3, 12, Reduced: Bcl-2	Liu et al. (2018)
		5 and 10 μM	Leukemia (CML)	K562 cell line	Induced: Caspase 3 and PARP1	Wei et al. (2013)
					Reduced: NR	
		2.5 and 5 μM	Colon	RKO cell line	Induced: Bax, activation of caspase 3 and -9. Accumulation of ROS	Zhang et al. (2013)
					Reduced: Bcl-2, MMP	
		7.5, 15, and 30 µM for cell line	Colon	HCT116 cell line	Induced: Bax, activation of caspase 3	Ren et al. (2021)
		50 mg/kg for animal model		Balb/c mice	Reduced: Bcl-2, MMP, and tumor size	
		10 and 20 μM	Gastric	BGC-823 and SGC-7901 cell lines	Induced: Bax	He et al. (2019)
					Reduced: Bcl-2	
		40 μM	Glioblastoma multiform	U 87 cell line	Induced: Bax, cytochrome C release, activation of caspase 3, and 9	Khan et al. (2012b)
					Reduced: Bcl-2	
		40 μM	Liver	HepG2 cell line	Induced: Bax, cytochrome C release, activation of caspase 3, 8, and 9, Bid cleavage	Lei et al. (2012), Khan et al. (2013)
					Reduced: Bcl-2, MMP	
		45 and 60 μM	Lung	A549 cell line	Induced: Bax, Bad activation of caspase 3 and 9	Maryam et al. (2017)
					Reduced: Bcl-2, XIAP, Survivin	
		4, 6, 8, and 10 μM	Melanoma	A375 and B16 cell lines	Induced: Bax, activation of caspase 3	Zhang et al. (2023a)
					Reduced: Bcl-2	
		5 and 7.5 μM	Multiple myeloma	OPM2 and IM-9 cell lines	Induced: Bax, activation of caspase 3 and 9	Yao et al. (2015)
					Reduced: Bcl-2, Survivin	
		8 and 12 μM	Oral	CAL27 and SCC9 cell lines	Induced: ROS	Zhang et al. (2023b)
					Reduced: MMP	
		4, 8, 10 μM for cell lines	Osteosarcoma	143B and MG63 cell lines	Induced: Bax, Bad activation of caspase 3, PARP	Yang et al. (2022a)
		15 and 25 mg/kg over 30 days for animal model		BALB/c mice	Reduced: Bcl-2, Tumor size	
		44.75 μM for alantolactone	Ovary	SKOV3 cell line	Induced: Bax, ROS	Alipour et al. (2022)
		30.76 μM for cisplatin			Reduced: Bcl-2, XIAP, cyclin D1	
		16.56 μM for ZnONPs				
		20 and 40 μM	Lung	SK-MES-1 cell line	Induced: Bax, activation of caspases 3, 8, and 9, PARP	Zhao et al. (2015)
					Reduced: Bcl-2	
13-O-acetylsolstitialin	*Centaurea cyanus*	10 and 100 μM	Breast	MDA-MB-231 and MCF-7 cell lines	Induced: Bax, ROS	Keyvanloo Shahrestanaki et al. (2019)
					Reduced: Bcl-2, MMP	
Ambrosin	*Hymenoclea salsola* and *Ambrosia maritima*	IC50 = 25 μM	Breast	MDA-MB-231 cell line	Induced: Bax, ROS	Fan et al. (2020)
					Reduced: Bcl-2, MMP	
		8, 32, and 64 μM	Breast	MDA-MB-231 cell line	Induced: Bax, ROS, activation of caspase 3 and 9	Meng and Shao (2021)
					Reduced: Bcl-2	
Antrocin	*Antrodia cinnamomea*	100 μM	Bladder	5637 cell line	Induced: Bax, Fas, FasL, DR5, activation of caspases 3, 8, and 9	Chiu et al. (2016)
					Reduced: Bcl-2, Bcl-xL	
		10 μM	Breast	MDA-MB-231 cell line	Induced: Bax, cytochrome C release, activation of caspase 3, Reduced: Bcl-2, Bcl-xL, survivin	Rao et al. (2011)
		5, 10 μM	Lung	H441 and H1975 cell lines	Induced: Bax, activation of caspase 3, Reduced: Bcl-2, MCL-1, survivin	Yeh et al. (2013)
		100 μM	Prostate	PC3-KD cell line	Induced: Bax, activation of caspases 3 and 9, PARP	Chen et al. (2018c)
					Reduced: Bcl-2	
Artemisinin	*Artemisia annua*	50 and 100 μM	Breast	MDA-MB-231 cell line	Induced: Bax	Guan and Guan (2020)
					Reduced: Bcl-2	
		20 μM for cell lines	Gallbladder	GBC-SD and NOZ cell lines	Induced: ROS, cytochrome C release, activation of caspase 3	Jia et al. (2016)
		100 mg/kg per day) over 30 days for animal model		BALB/c nude mice	Reduced: MMP	
		2.5, 5, 10, 20 and 40 μg/ml for cell lines	Glioma	C6 cell line, Rat model	Induced: Bax, Caspase 3	Chen et al. (2018a)
		10 mg/kg per day) over 10 days for animal model			Reduced: Bcl-2	
		5, 25 and 50 μM ART and DHA for cell lines	Liver	HepG2 and Hep3B cell lines	Induced: Bax, activation of caspase 3, PARP	Hou et al. (2008)
		50 and 100 mg ART and DHA/kg/d, 5 days/week for 4 weeks animal model		BALB/c mice	Reduced: Bcl-2, Tumor size	
		100 and 150 μM	Lung	A549 cell line	Induced: Bax, activation of caspase 3, cytochrome C release, ROS	Ganguli et al. (2014)
					Reduced: Bcl-2	
		50 μM	Breast	T-47D cell line	Induced: Bax, activation of caspase 3 and 7	Hassani et al. (2022)
					Reduced: Bcl-2	
		300 μM	Neuroblastoma	SK-N-AS, SK-N-DZ and SHEP1 cell lines	Induced: NR	Zhu et al. (2014)
					Reduced: NR	
Deoxoartemisinin trimer	*Artemisinin derivative*	25 μM	Oral	YD-10B cell line	Induced: Activation of caspase 3	Nam et al. (2007)
					Reduced: NR	
Brevilin A	*Centipeda minima*	10 and 15 μM	Breast	MCF-7 cell line	Induced: Bax, Bak activation of caspase 9 and PARP, accumulation of ROS	Saleem et al. (2020)
					Reduced: Bcl-2, XIAP	
		1, 2, and 4 μM for cell line	Colon	CT26 cell line	Induced: Bax, Bak activation of caspase 9 and PARP, accumulation of ROS	You et al. (2018)
		5 mg/kg per day) over 14 days for animal model		BALB/c mice	Reduced: Bcl-2, XIAP, tumor size	
		50 and 100 μM	Gastric	AGS cell line	Induced: Bax, Bak activation of caspase 9 and PARP, accumulation of ROS	Lee et al. (2022)
					Reduced: Bcl-2, XIAP, tumor size	
		10 and 20 μM	Glioblastoma	U87 cell line	Induced: Bax, cytochrome C release, activation of caspase 9 and 3, PARP, accumulation of ROS	Wang et al. (2018b)
					Reduced: Bcl-2, XIAP, MMP	
		20 and 30 μM	Lung	A549 and NCI-H1650 cell lines	Induced: Bax, activation of caspase 3, PARP, accumulation of ROS	Khan et al. (2020)
					Reduced: Bcl-2, MMP	
		10 and 20 μM for cell line	Nasopharynx	CNE-2 cell line	Induced: Bax, activation of caspase 9, PARP	Liu et al. (2019a)
		10 and 20 mg/kg per day) over 16 days for animal model		BALB/c mice	Reduced: Bcl-2, tumor size	
		3 μM	Melanoma	A375 and A2058 cell lines	Induced: Activation of caspases 3, 7, 8, and 9, PARP	Su et al. (2020)
					Reduced: Bcl-xL, Mcl-1	
Bigelovin	*Inula helianthus aquatic*	5 μM	Colorectal	HT-29 and HCT 116 cell lines	Induced: Activation of caspases 3, and 9, PARP	Feng et al. (2021)
					Reduced: Bcl2	
		1.8, 3.6, and 5.4 μM for HT-29 cell line	Colorectal	HT-29 and HCT 116 cell lines	Induced: DR5, DcR2, Activation of caspases 3 and 7, PARP, accumulation of ROS	Li et al. (2017)
		1.4, 2.8, and 4.2 μM for HCT 116 cell line		BALB/c mice	Reduced: Bax, Bcl2, caspases 8 and 9, tumor size	
		20 mg/kg over 7 days for animal model				
		2.5, 5, and 10 μM for cell lines	Liver	HepG2 and SMMC-7721cell lines	Induced: Bax, activation of caspases 3 and 9, PARP, accumulation of ROS	Wang et al. (2018a)
		5, 10, and 20 mg/kg over 21 days for animal model		BALB/c mice	Reduced: Bcl2, tumor size	
Britannin	*Inula aucheriana*	30 μM for AsPC-1 and 40 μM for Panc-1	Pancreas	AsPC-1 and Panc-1 cell lines	Induced: Bax, cytochrome C release, activation of caspases 3 and 9, PARP, accumulation of ROS	Moeinifard et al. (2017)
					Reduced: Bcl-2	
		1, 3 and 5 μM	Leukemia (ALL)	NALM-6 cell line	Induced: Bax, accumulation of ROS	Mohammadlou et al. (2021)
					Reduced: XIAP	
		3 and 5 μM	Leukemia (ALL)	MOLT-4 cell line	Induced: Bax, Bim, activation of caspases 3, accumulation of ROS	Mohammadlou et al. (2022b)
					Reduced: Bcl-2	
		10–100 μM	Breast	MCF-7 and MDA-MB-468 cell lines	Induced: Bax, cytochrome C release, activation of caspases 3 and 9, accumulation of ROS	Hamzeloo-Moghadam et al. (2015)
					Reduced: Bcl-2, MMP	
		5 and 7 μM	Leukemia (AML and CML)	U937 and K562 cell lines	Induced: BAX, BAD, and BID	Mohammadlou et al. (2022a)
					Reduced: BCL-2, BCL-XL, and MCL-1	
		20, 40, and 80 μM for cell line	Liver	HepG2 cell line, BALB/c mice	Induced: Bax, cytochrome C release, activation of caspases 3 and 9, accumulation of ROS	Cui et al. (2018b)
		7.5, 15, and 30 mg/kg over 21 days for animal model			Reduced: Bcl-2, MMP, tumor size	
		10 μM for cell line	Prostate	PC-3 cell line	Induced: Bax	Zeng et al. (2020)
		5 and 10 mg/kg over 25 days for animal model		BALB/c mice	Reduced: Bcl-2, tumor size	
Costunolide	*Saussurea lappa*	10 μM	Neuroblastoma	IMR-32 cell line	Induced: Activation of caspase 7, PARP	Tabata et al. (2015)
					Reduced: NR	
		15 μM	Breast	MDA-MB-231 cell line	Induced: Fas, Activation of caspases 3 and 8, PARP	Choi et al. (2012)
					Reduced: NR	
		80 μM	Esophagus	Eca-109 cell line	Induced: Bax, Activation of caspases 3, PARP, accumulation of ROS	Hua et al. (2016a)
					Reduced: Bcl-2, MMP	
		20 and 40 μM	Gastric	HGC-27 and SNU-1 cell lines	Induced: Bax, Bak, Activation of caspases 3, PARP, accumulation of ROS	Xu et al. (2021)
					Reduced: Bcl-2	
		15, 20, and 25 μM for cell line	Gastric	BGC-823 cell line, BALB/c mice	Induced: Bax, Bak, activation of caspases 3, 9 and 7, accumulation of ROS, PARP	Yan et al. (2019)
		50 mg/kg over 36 days for animal model			Reduced: Bcl-2, MMP, tumor size	
		10 μM for cell line	Leukemia	U937 cell line, BALB/c mice	Induced: Accumulation of ROS	Choi and Lee (2009)
		7.5, 15, and 30 mg/kg over 36 days for animal model			Reduced: Bcl-2, tumor size	
		20 and 30 μM	Lung	A549 cell line	Induced: Bax, Activation of caspases 3 and 9, PARP, release of cytochrome C, accumulation of ROS	Wang et al. (2016)
					Reduced: Bcl-2	
		40 and 80 μM	Lung	SK-MES-1 cell line	Induced: Bax, Activation of caspase 3, PARP	Hua et al. (2016b)
					Reduced: Bcl-2	
		32 and 39 μM	Oral	CAL 27 cell line	Induced: Bak, Activation of caspases 3 and 6	Shams et al. (2023)
					Reduced: Bcl-2L1	
		20 μM	Ovary	MPSC1 and SKOV3 cell lines	Induced: Activation of caspases 3, 8 and 9, accumulation of ROS	Yang et al. (2011)
					Reduced: Bcl-2	
		Costunolide (20 μM) + doxorubicin (200 μM)	Prostate	PC-3 and DU-145cell lines	Induced: Bax, Bak, activation of caspases 3 and 9, accumulation of ROS	Chen et al. (2017)
					Reduced: Bcl-2, Bcl-xL	
		20 and 40 μM	Kidney	769-P cell line	Induced: Bax, release of cytochrome C, activation of caspases 3 and 9, accumulation of ROS	Fu et al. (2020)
					Reduced: Bcl-2, MMP	
		20 and 30 μM	Osteosarcoma	U2OS cell line	Induced: Bax, release of cytochrome C, activation of caspases 3 and 9, accumulation of ROS	Zhang et al. (2016)
					Reduced: Bcl-2, MMP	
		0.8 μM	Skin	A431 cell line	Induced: Bax, activation of caspase 3, PARP	Lee et al. (2021)
					Reduced: Bcl-2, Bcl-xL	
Cynaropicrin	*Saussurea lappa*	10 μM	Leukemia	U937, Eol-1, and Jurkat T cell lines	Induced: accumulation of ROS	Cho et al. (2004)
					Reduced: NR	
		25 μM	Glioblastoma	U-87 MG cell line	Induced: Activation of caspases 3 and 9, release of cytochrome C, accumulation of ROS	Rotondo et al. (2022)
					Reduced: MMP	
		10, 20, 40 μM	Cervix	Hela cell line	Induced: Activation of caspase 3, accumulation of ROS	Liu et al. (2019b)
					Reduced: NR	
		5 and 10 μM for cell lines	Neuroblastoma	SK-N-BE (2) and SH-SY5Y cell lines	Induced: Bax, activation of caspase 3, PARP	Yang et al. (2022b)
		2.5 mg/kg over 24 days for animal model		BALB/c mice	Reduced: Bcl-2, tumor size	
Dehydrocostus lactone	*Saussurea lappa*	1.1 and 1.7 μg/mL for cell lines	Breast	MCF-7 and MDA-MB-231 cell lines	Induced: Bax	Peng et al. (2017)
		20 mg/kg over 31 days for animal model		BALB/c mice	Reduced: Bcl-2, p-BID tumor size	
		4 and 8 μg/mL	Breast	MCF-7 and MDA-MB-231 cell lines	Induced: Bax, Bad, activation of caspase 9, translocation of AIF and EndoG	Kuo et al. (2009)
					Reduced: Bcl-2, Bcl-xL	
		5 and 10 μg/mL	Leukemia	K562 cell line	Induced: Bax	Cai et al. (2019)
					Reduced: Bcl-2	
		8 and 12 μM	Leukemia	K562 cell line	Induced: Bax, accumulation of ROS	Cai et al. (2017)
					Reduced: Bcl-2, Bcl-xL, Mcl-1, MMP	
		8, 16, or 32 μM for cell lines	Esophagus	Eca109 and KYSE150 cell lines	Induced: Bax, activation of caspases 3 and 9, PARP, accumulation of ROS	Peng et al. (2022)
		20, 40 mg/kg over 28 days for animal model		BALB/c mice	Reduced: Bcl-2, tumor size	
		10, 20 and 30 μM	Glioblastoma	U87 cell line	Induced: Bax, activation of caspases 3 and 9, release of cytochrome C	Wang et al. (2017)
					Reduced: Bcl-2	
	*Saussurea costus*	2, 4 and 6 μg/mL for cell lines	Laryngeal	Hep-2 and TU212 cell lines	Induced: Bax, activation of caspases 3 and 9, PARP	Zhang et al. (2020)
		10 and 15 mg/kg over 28 days for animal model		BALB/c mice	Reduced: Bcl-2, tumor size	
	*Aucklandia lappa Decne*	10 and 15 μM for cell lines	Liver	HepG2 and SK-HEP-1 cell lines	Induced: Bax, PARP	Tian et al. (2023)
		10 and 20 mg/kg over 22 days for animal model		BALB/c mice	Reduced: Bcl-2, tumor size	
	*Saussurea lappa*	15 and 30 μM	Liver	HepG2 and PLC/PRF/5 cell lines	Induced: Bax, Bak, translocation of AIF and EndoG	Hsu et al. (2009b)
					Reduced: Bcl-2, Bcl-xL	
	*Glossogyne Tenuifolia*	10 and 20 μg/mL	Lung	A549 cell line	Induced: Activation of caspases 3 and 9	Hsu et al. (2009a)
					Reduced: NR	
	*Saussurea lappa*	1 μM	Lung	A549 and H460 cell lines	Induced: Activation of caspases 3 and 9, PARP	Sheng et al. (2018)
					Reduced: Bcl-2	
		100 μM	Ovary	SK-OV-3 cell line	Induced: Bax, activation of caspase 3, release of cytochrome C, PARP	Choi and Ahn (2009)
					Reduced: Bcl-2	
		2 mg/L	Prostate	DU145 cell line	Induced: Bax, Bak, Bok, Bik,t-Bid, Bmf, activation of caspase 3, 7, 8, 9, release of cytochrome C, PARP	Kim et al. (2008)
					Reduced: Bcl-xL	
		7.41, 6.17, 8.33 μg/L	Sarcoma	SW-872, SW-982, and TE-671 cell lines	Induced: Activation of caspase 3 and 7, PARP	Kretschmer et al. (2012)
					Reduced: NR	
Deoxyelephantopin	*Elephantopus scaber*	50 μM	Liver	HepG2 cell line	Induced: Bax, activation of caspase 3, release of cytochrome C, accumulation of ROS, PARP	Mehmood et al. (2017b)
					Reduced: Bcl-2, MMP	
		2 μg/mL for cell line	Breast	TS/A cell line	Induced: Bax, activation of caspases 3, 7, 6 8 and 9, accumulation of ROS, PARP	Huang et al. (2010)
		10 mg/kg over 28 days for animal model		BALB/c mice	Reduced: Tumor size	
		4.14 μM	Cervix	SiHa cell line	Induced: Bax, activation of caspases 3, 7, 8, and 9, accumulation of ROS, PARP	Farha et al. (2014)
					Reduced: Bcl-2, Bcl-xL	
		10 and 20 μM	Cervix	HeLa cell line	Induced: Activation of caspases 3 and 9, PARP	Zou et al. (2008)
					Reduced: NR	
		2, 5, and 10 μM for cell lines	Colorectal	HCT116 and sw620 cell lines	Induced: Activation of caspase 3 and PARP	Ji et al. (2022)
		30 mg/kg over 30 days for animal model		BALB/c mice	Reduced: Bcl-2, tumor size	
		1.5 and 3 μg/mL	Colorectal	HCT116 cell line	Induced: Activation of caspase 3 and PARP	Chan et al. (2016)
					Reduced: NR	
		11.6, 23.2 and 46.5 μg/mL	Nasopharynx	CNE cell line	Induced: Bax, Bad, Bok, Bmf, and PUMA, FasL, t-BID, release of cytochrome C, activation of caspases 3, 7, 8, 9, and 10	Su et al. (2011)
					Reduced: Bcl-2, Bcl-xL, MMP	
		4, 8 and 16 μM for MG-63, 8, 16, 32 μM for U2OS	Osteosarcoma	MG-63 and U2OS cell lines	Induced: Bax, activation of caspases 3, 9, and PARP, accumulation of ROS	Zou et al. (2017)
					Reduced: Bcl-2	
		30 and 50 μM for BxPC-3, 40 and 60 μM for CFPAC-1	Colorectal	BxPC-3 and CFPAC-1 cell lines	Induced: Bax, activation of caspase 3 and 9, release of cytochrome C	Ji et al. (2020)
		10 mg/kg over 24 days for animal model		BALB/c mice	Reduced: Bcl-2, tumor size	
Ergolide	*Inula britannica*	4 and 6 μM	Leukemia	Nalm6 and MOLT-4 cell lines	Induced: Bax, Bim, activation of caspase 3 and PARP	Song et al. (2005)
					Reduced: Bcl-2, XIAP cIAP1	
		4 and 6 μM	Colorectal	Nalm6 and MOLT-4 cell lines	Induced: Bax, Bim, activation of caspase 3 and PARP, release of cytochrome C	Song et al. (2005)
					Reduced: Bcl-2, XIAP cIAP1	
Eupalinolide J	*Eupatorium lindleyanum*	10 and 20 μM	Prostate	PC-3 and DU-145 cell lines	Induced: Activation of caspases 3 and 9	Wu et al. (2020)
					Reduced: MMP	
Eupalinolide O	*Eupatorium lindleyanum*	5 and 10 μM for cell lines	Breast	MDA-MB-231 and MDA-MB-435 cell lines	Induced: Bax, activation of caspases 3 and 9, PARP, accumulation of ROS	Zhao et al. (2022)
		15 and 30 mg/kg over 20 days for animal model		BALB/c mice	Reduced: Bcl-2, MMP, tumor size	
Eupalinolide O	*Eupatorium lindleyanum*	4 and 8 μM	Breast	MDA-MB-468 cell line	Induced: Bax, Bad, activation of caspases 3 and 9, PARP	Yang et al. (2016)
					Reduced: Bcl-2, Bcl-xL	
Gaillardin	*Inula oculus-christi*	10 and 100 μM	Breast	MDA-MB-468 and MCF-7 cell lines	Induced: Bax, activation of caspases 3 and 9, accumulation of ROS	Fallahian et al. (2015)
					Reduced: Bcl-2, MMP	
		6 μM	Leukemia (ALL)	NALM-6 and MOLT-4 cell lines	Induced: Bax, caspase 3	Karami et al. (2020)
					Reduced: Bcl-2	
		7 μM	Leukemia (APL)	NB4 cell line	Induced: Bax, Bad	Sayyadi et al. (2021)
					Reduced: Bcl-2	
		40 μM	Gastric	AGS and MKN45 cell lines	Induced: Bax, caspase 3	Sayyadi et al. (2021)
					Reduced: Bcl-2	
Helenalin	*Arnica montana* and *Arnica chamissonis ssp. Foliosa*	10–50 μM	Leukemia	Jurkat T cell line	Induced: Activation of caspases 3 and 8, release of cytochrome C	Dirsch et al. (2001)
					Reduced: MMP	
Isoalantolactone	*Inula helenium L*	5, 10, and 20 μM for cell lines	Colorectal	HCT116 and SW620 cell lines	Induced: Bax, activation of PARP	Li et al. (2022)
		10 and 20 mg/kg over 22 days for animal model		BALB/c mice	Reduced: Bcl-2, Bcl-xL, Mcl-1, tumor size	
		20 and 40 μM	Gastric	SGC-7901 cell line	Induced: Bax, activation of caspase 3	Rasul et al. (2013b)
					Reduced: Bcl-2, MMP	
		10, 20, and 30 μM for cell line	Glioblastoma	U87 cell line	Induced: Bax, release of cytochrome C, activation of caspases 3 and 9, PARP	Xing et al. (2019)
		30 mg/kg over 15 days for animal model		BALB/c mice	Reduced: Bcl-2, tumor size	
		25 and 50 μM	Head and neck	UM-SCC-10A cell line	Induced: Bax, release of cytochrome C, activation of caspase 3	Wu et al. (2013)
					Reduced: Bcl-2, MMP	
		10, 15 and 20 μM	Leukemia	K562/A02 cell line	Induced: Bax, release of cytochrome C, activation of caspases 3 and 9, PARP, accumulation of ROS	Cai et al. (2014)
					Reduced: Bcl-2	
		1, 3 and 5 μM	Liver	Hep3B cell line	Induced: Bax, t-Bid, DR4, DR5, Fas, release of cytochrome C, activation of caspases 3, 8 and 9, PARP, accumulation of ROS	Kim et al. (2021)
					Reduced: Bcl-2	
		20 and 40 μM	Lung	SK-MES-1 cell line	Induced: Bax, activation of caspase 3 and PARP, accumulation of ROS	Jin et al. (2017)
					Reduced: Bcl-2, MMP	
		20 and 40 μM	Osteosarcoma	U2OS cell line	Induced: Bax, DR5, FADD, TRADD, activation of caspases 3 and 8, PARP, accumulation of ROS	Di et al. (2014)
					Reduced: Bcl-2, MMP	
		10, 20, 30, 40, and 50 μM	Ovary	SKOV-3 and OVCAR-3 cell lines	Induced: Bax, activation of caspase 3 and PARP, accumulation of ROS	Xie et al. (2023)
					Reduced: Bcl-2	
		20 and 40 μM	Pancreas	PANC-1 cell line	Induced: Bax, release of cytochrome C, activation of caspase 3, accumulation of ROS	Khan et al. (2012a)
					Reduced: Bcl-2, MMP	
		10, 20 and 40 μM for cell lines	Pancreas	PANC-1, AsPC-1, and BxPC-3 cell lines	Induced: Bax, activation of caspase 3, Reduced: Tumor size	Zhang et al. (2021a)
		0.5 mg/kg over 28 days for animal model		BALB/c mice		
		10, 20 and 40 μM	Prostate	PC-3 and DU145 cell lines	Induced: Bax, activation of caspases 3 and 9	Chen et al. (2018b)
					Reduced: Bcl-2	
		20 and 40 μM	Prostate	PC-3 cell line	Induced: Bax, activation of caspase 3, accumulation of ROS	Rasul et al. (2013a)
					Reduced: Bcl-2, MMP	
Isocostunolide	*Inula helenium*	1.25, 2.5, 5, and 7.5 μM	Melanoma	A2058 cell line	Induced: Bax, Fas activation of caspases 3 and 8, PARP, release of cytochrome C, accumulation of ROS	Chen et al. (2007)
					Reduced: Bcl-2, Bid	
Isodeoxyelephantopin	*Elephantopus scaber Linn*	2 μM	Leukemia	KBM-5 cell line	Induced: Activation of PARP	Ichikawa et al. (2006)
					Reduced: IAP1/2, Bcl-2, Bcl-xL, Bfl-1/A1, TRAF1, FLIP, and survivin	
		1, 2.5 and 5 μM	Breast	MDA-MB-231 cell line	Induced: Activation of caspases 7 and 9, PARP	Verma et al. (2019)
					Reduced: Bcl-2, Bcl-xL, MMP	
		10.46 μg/mL for A549 cells and 1.3 μg/mL for T47D cells	Breast and Lung	A549 and T47D cell lines	Induced: Activation of caspases 7 and 9, PARP	Kabeer et al. (2014)
					Reduced: Bcl-2, Bcl-xL, MMP	
Janerin	*Centaurothamnus maximus*	12.5, 5, and 10 μM	Leukemia (AML)	THP-1 cell line	Induced: Bax, activation of caspase 3 and PARP	Ahmed et al. (2021)
					Reduced: Bcl-2	
Lactucopicrin	*Lactuca virosa*	12, 25, and 50 μM	Osteosarcoma	Saos-2 cell line	Induced: Bax	Meng et al. (2019)
					Reduced: Bcl-2	
		7.5, 15, and 30 μM	Skin	SKMEL-5 cell line	Induced: Bax	Zhang et al. (2018a)
					Reduced: Bcl-2	
		7.5 μM	Glioblastoma	U87 Mg cell line	Induced: Bax	Rotondo et al. (2020)
					Reduced: Bcl-2	
Parthenolide	*Tanacetum parthenium*	1–10 μM	Leukemia	NCI-H929, Farage, Raji, 697, KOPN-8, CEM, MOLT-4 cell lines	Induced: Fas-L, activation of caspase 3, accumulation of ROS	Jorge et al. (2023)
					Reduced: MMP	
		2.5, 5, 7.5 and 10 μM	Bladder	5637 cell line	Induced: PARP	Cheng and Xie (2011)
					Reduced: Bcl-2	
		50 μM	Breast	231MFP and HCC38 cell lines	Induced: Activation of caspases 3 and 7	Berdan et al. (2019)
					Reduced: NR	
		9 and 15 μM	Breast	MDA-MB-468 cell line	Induced: Bax, activation of caspase 3	Ghorbani-Abdi-Saedabad et al. (2020)
					Reduced: Bcl-2	
		4.5–11.5 μM	Breast and Cervix	SiHa and MCF-7 cell lines	Induced: Bax, activation of caspase 3	Al-Fatlawi et al. (2015)
					Reduced: Bcl-2	
		6 μM	Cervix	HeLa cell line	Induced: Bax, activation of caspase 3	Jeyamohan et al. (2016)
					Reduced: Bcl-2	
		10 and 20 μM	Cholangiocarcinoma	SCK, JCK, Cho-CK, and Choi-CK cell lines	Induced: Bax, Bak, tBid	Kim et al. (2005)
					Reduced: Fas, Fas-L	
		5, 10 and 20 μM	Colorectal	SW620 cell line	Induced: Activation of caspase 3	Liu et al. (2017b)
					Reduced: Bcl-2, Bcl-xL	
		5, 10, 20 and 40 μM for cell lines	Colorectal	HT-29, SW620 and LS174T cell lines BALB/c mice	Induced: Bax, release of cytochrome C, activation of caspase 3, PARP	Kim et al. (2012)
		4 mg/kg over 30 days for animal model			Reduced: Bcl-2, tumor size	
		20 μM	Glioblastoma	U-87 MG cell line	Induced: Activation of caspases 3 and 7	Anderson and Bejcek (2008)
					Reduced: NR	
		2.5 and 5 μM for cell lines	Liver	HepG2, MHCC 97H, Huh7 and H22 cell lines	Induced: Bax, activation of caspase 3	Yi et al. (2022)
		25 and 50 mg/kg + arsenic trioxide (25 mg/kg) over 28 every other day for animal model		BALB/c mice	Reduced: Bcl-2, tumor size	
		10, 100 and 500 μM	Lung	A549 cell line	Induced: Activation of caspases 3 and 9	Fang et al. (2010)
					Reduced: NR	
		6 and 12 μM	Melanoma	A375 cell line	Induced: Activation of caspase 3, accumulation of ROS	Lesiak et al. (2010)
					Reduced: MMP	
		2.5, 5, 7.5 and 10 μM	Ovary	OVCAR-3 and SK-OV-3 cell lines	Induced: Bax, activation of caspases 3, 8 and 9, PARP, accumulation of ROS	Kwak et al. (2014)
					Reduced: Bcl-2, Bcl-xL, Bid, Survivin	
		2.5, 5 and 10 μM	Pancreas	Panc-1 and BxPC3 cell lines	Induced: Activation of caspase 3 and PARP	Liu et al. (2017a)
					Reduced: NR	
Santamarine	*Magnolia grandiflora and Ambrosia confertiflora*	50 and 100 μM	Liver	HepG2 cell line	Induced: Bax, Bad, release of cytochrome C, activation of Bid and caspases 3, 8 and 9, PARP, accumulation of ROS	Mehmood et al. (2017a)
					Reduced: Bcl-2, MMP	
		40 and 60 μM	Lung	A549 cell line	Induced: Bax, activation of caspase 3, accumulation of ROS	Wu et al. (2017)
					Reduced: Bcl-2, MMP	
		10, 25, or 50 μM	Cervix	A549 cell line	Induced: Activation of caspase 3, accumulation of ROS	Zhang et al. (2021b)
					Reduced: NR	
Sesquiterpene lactone 3	*Artemisia argyi*	5, 10, or 20 μM	Gastric	AGS and MGC803 cell lines	Induced: Activation of caspase 3 and PARP	Zhang et al. (2018b)
					Reduced: NR	
Uvedafolin	*Smallanthus sonchifolius*	3 μM	Cervix	HeLa cell line	Induced: Activation of caspases 3, 7 and 9, release of cytochrome C	Kitai et al. (2017)
					Reduced: MMP	
Vernolactone	*Vernonia Zeylanica*	10 μM	Breast	MCF -7, MDA-MB-231, SKBR-3 cell lines	Induced: Bax, activation of caspases 3 and 7	Mendis et al. (2019)
					Reduced: Survivin	
		2 and 4 μg/mL	Embryonic carcinoma	NTERA-2 stem-like cell	Induced: Activation of caspases 3 and 7	Abeysinghe et al. (2019)
					Reduced: Survivin	

NR, Not reported; MMP, Mitochondrial membrane potential; ROS, Reactive oxygen species; PARP, Poly (ADP-ribose) polymerase.

### 4.2 Ambrosin

In a study on a human drug-resistant breast cancer cell line MDA-MB-231, the anti-cancer activity of ambrosin has been evaluated. Ambrosin is a sesquiterpene lactone found in *Hymenoclea salsola* and *Ambrosia maritime*. The data shows that ambrosin induces apoptosis of MDA-MB-231 cells by increasing Bax and decreasing Bcl-2 expression levels. The IC_50_ value of ambrosin was calculated as 25 µM. However, ambrosin treatment at a concentration of 50 µM increased apoptotic MDA-MB-231 cells from 3.5% in the controls to 56%. This sesquiterpene lactone decreased MMP and increased the levels of ROS in MDA-MB-231 cells in a dose-dependent manner. Moreover, ambrosin had very low toxic effects on the MCF-12A normal breast cells (Fan et al., 2020). In a similar study, ambrosin significantly enhanced the apoptotic cell death in MDA-MB-231 cells in a dose- and time-dependent manner. It also led to the enhanced expression levels of caspase-3, caspase-9, and Bax with decreased levels of Bcl-2 in MDA-MB-231 cells at concentrations more than 8 µM (Meng and Shao, 2021).

### 4.3 Antrocin

Antrocin is a sesquiterpene lactone derived from *Antrodia cinnamomea* with different biological activities. A study by Chiu et al. (2016) unraveled that antrocin could stimulate both intrinsic and extrinsic pathways of apoptosis in the human bladder cancer 5637 cell line. Their data evidenced the upregulation of Fas, FasL, DR5, and Bax with enhanced activity of caspases 3, 8, and 9. Antrocin also decreased the protein expression of Bcl-2 and Bcl-xL. Rao et al. (2011) investigated the anticancer effects of antrocin on triple-negative breast cancer cells MDA-MB-231. They found that antrocin promoted apoptosis of these cancer cells by a mechanism that leads to the activation of caspase-3 and PARP enzymes. Antrocin also increased the protein expression of pro-apoptotic factor Bax, enhanced the release of cytochrome C, and suppressed the expression of anti-apoptotic proteins Bcl-2, Bcl-xL, and survivin in a time- and dose-dependent manner in MDA-MB-231 cells. In another *in-vitro* and *in-vivo* study on breast cancer models, the authors asserted that antrocin treatment triggered apoptosis in MCF-7 and MDA-MB-231 cell lines by a mechanism that targets β-catenin, Akt, and Notch. Hey also revealed a significant suppressing effect of antrocin on the MDA-MB-231 xenograft model. However, they have not evaluated any apoptosis marker (Chen et al., 2019). Yeh et al. (2013) showed that antrocin suppressed the proliferation of two non-small-cell lung cancer cells, H441 and H1975. Antrocin also induced apoptosis in these cells by activating caspase 3, increasing the expression of Bax, and decreasing the levels of Bcl2, MCL-1, and survivin in H441 and H1975 cell lines. In prostate cancer cell line PC3-KD, antrocin initiates apoptotic cell death mechanism through modulating different apoptosis markers. This sesquiterpene lactone led to elevated levels of cleaved forms of caspase 3, caspase 9, and PARP. It further upregulated Bax and downregulated Bcl-2 to trigger apoptosis in PC3-KD cells (Chen Y. A. et al., 2018).

### 4.4 Artemisinin

Artemisinin is a sesquiterpene lactone isolated from *Artemisia annua* L. with apoptosis-inducing effects on T-cell leukemia cell line Molt-4 (Singh and Lai, 2004). Artemisinin shows pro-apoptotic effects on cisplatin-resistant MDA-MB-231 breast cancer cells. It acts by downregulating Bcl-2 and upregulating Bax protein expressions (Guan and Guan, 2020). Primary gallbladder cancer cell lines (GBC-SD and NOZ) undergo apoptosis when treated with artemisinin. Anti-proliferative activity of artemisinin was also revealed in an *in-vitro* and an *in-vivo* xenograft model of gallbladder cancer. Western blot analysis demonstrated that artemisinin treatment led to cytochrome C release into the cytoplasm and activated caspase-3 in both cell lines. Artemisinin resulted in G1-phase arrest, promoted ROS generation, and decreased MMP in these gallbladder cancer cells (Jia et al., 2016). Chen J. et al. (2018) treated C6 glioma cells and a rat C6 brain-glioma model with artemisinin to evaluate the anti-tumor activity of artemisinin *in-vitro* and *in-vivo*. They asserted that artemisinin had decreased Bcl-2 protein levels and increased Bax and caspase 3 expressions in C6 cells but their western blotting results are not clearly evident and do not show significant changes. However, the expression of these factors has been clearly changed in the rat model of disease. In human hepatocarcinoma cell lines (HepG2 and Hep3B), artemisinin and dihydroartemisinin triggered apoptosis through increasing the ratio of Bax to Bcl-2 and activating caspase 3 and PARP in line with the downregulation of mouse double minute 2 (MDM2). In *in-vivo* models of HepG2 and Hep3B xenograft tumors, both compounds suppressed tumor size and altered the expression of the above-mentioned apoptosis markers similar to *in-vitro* models (Hou et al., 2008). Artemisinin treatment can induce autophagy in non-small lung carcinoma (A549) cells at the early stage of treatment. This compound also induces apoptosis in A549 cells via a mitochondrial-mediated pathway by decreasing the expression of Bax, increasing the expression of Bcl-2 and cytochrome C leading to the activation of caspase 3. ROS production is another mechanism of apoptosis induction by artemisinin. Pre-treatment with chloroquine synergistically enhanced the apoptosis-inducing activity of artemisinin in A549 cells (Ganguli et al., 2014). A combination of artemisinin and metformin has been reported to induce cell cycle arrest in T-47D breast cancer cells. This combined treatment triggered apoptosis in these cancer cells through enhancing Bcl-2, suppressing Bax and survivin as well as the activation of caspase 7 and caspase 3. Nano-formulated forms of this combination even had stronger anti-proliferative activity in comparison to free forms (Hassani et al., 2022). Zhu et al. (2014) studied the pro-apoptotic effect of artemisinin on human neuroblastoma cell lines SK-N-AS, SK-N-DZ, and SHEP1 by flow cytometry. However, they have not checked the expression of apoptosis markers to further support flow cytometry data. Nam et al. (2007) demonstrated that deoxoartemisinin trimer, an artemisinin derivative, showed strong antiproliferative and apoptosis-inducing effects on oral cancer cell line YD-10B. They clarified that this derivative induced apoptosis by acting on a mechanism that involves caspase 3 activation but no other intrinsic or extrinsic markers of apoptosis were checked.

### 4.5 Brevilin A

Brevilin A, a sesquiterpene lactone from *Centipeda minima*, has been unraveled to promote apoptosis in MCF-7 breast carcinoma cells by enhancing G2/M phase cell cycle arrest. Induction of apoptosis by Brevilin A led to the augmentation of Bax and Bak, formation of ROS, diminution of Bcl-2 and XIAP, and activation of caspase 9 and PARP. However, no data were reported on the activation of caspase 3, as the leading marker of apoptosis induction (Saleem et al., 2020). You et al. (2018) evaluated the anti-tumor effects of Brevilin A on *in-vivo* and *in-vitro* models of colon adenocarcinoma CT26 cells. The results showed that Brevilin A induces apoptosis in these cancer cells by increasing ROS formation and decreasing MMP. Apoptosis induction by Brevilin A was identified by the formation of activated forms of caspase 8, caspase 9, and caspase 3 together with increased expression of Bax and decreased expression of Bcl-2. Their data also indicated that Brevilin A has positive effects on the induction of autophagy. The results of the *in-vivo* study indicated the growth-inhibitory activity of Brevilin A by triggering apoptosis and autophagy in tumor tissues. Lee et al. (2022) studied the effects of brevilin A on human gastric cancer (AGS) cells. They observed that CMX and brevilin A triggered apoptotic cell death AGS cells by increasing the activated forms of caspase 8 and caspase 3, reducing Bcl-2, and inducing the expression of Bax. In U87 glioblastoma cells, brevilin A reduced the proliferation and induced apoptotic cell death in a dose-dependent manner. The mechanism of brevilin A to stimulate apoptosis in U87 cells involved oxidative stress induction, Bax upregulation, Bcl-2 downregulation, MMP diminution, cytosolic cytochrome C accumulation, XIAP reduction, and increased expression of activated forms of caspase 9 and caspase 3, and PARP (Wang J. et al., 2018). Brevilin A inhibits proliferation and promotes morphological changes in two non-small cell lung cancer cells (A549 and NCI-H1650). Moreover, this sesquiterpene lactone upregulates Bax and downregulates Bcl-2 with no significant effect on XIAP protein expression. It also disrupts MMP and increases ROS levels in A549 and NCI-H1650 together with increased expression of cleaved forms of caspase 3 and PARP enzymes (Khan et al., 2020). Brevilin A upregulated Bax, cleaved caspase 9, and PARP along with a significant downregulation in the levels of Bcl-2 in human nasopharyngeal carcinoma cell line CNE-2 to induce cell cycle arrest at the G2/M phase. In the xenograft model of nude mice, brevilin A suppressed the growth of tumor tissue (Liu R. et al., 2019). This phytochemical suppressed the proliferation and stimulated apoptosis in melanoma cell lines A375 and A2058. Its pro-apoptotic activity was shown by activating caspases 3, 7, 8, and 9 together with the cleavage of PARP enzyme and decreased expression of anti-apoptotic proteins Bcl-xL and Mcl-1 (Su et al., 2020).

### 4.6 Bigelovin

Bigelovin is a sesquiterpene lactone isolated from *Inula helianthus aquatic*. This phytochemical has been shown to reduce the viability of human colon cancer cells and induce apoptosis of these cancer cells by a mechanism involving the inhibition of NF-κB downstream genes (Feng et al., 2021). Li, et al. described the anti-colorectal cancer effects of bigelovin *in-vitro* and *in-vivo*. Bigelovin induced apoptosis in HT-29 and HCT 116 cells by causing G2/M cell cycle arrest, activating caspases 3 and 7 and PARP, inducing DNA damage, upregulating DR5 or DcR2, and generating ROS. However, it had no remarkable effect on caspase 9 activation or showed a reducing effect on caspase 8, Bax, and Bcl-2. These data suggest that bigelovin may act on the extrinsic pathway to kill colorectal cancer cells. The growth xenograft model of the HCT116 tumor was reversed after bigelovin treatment. The results of the *in-vivo* study also confirmed the induction of the extrinsic pathway by bigelovin (Li et al., 2017). Intravenous injection of bigelovin at a range dose of 0.3–3 mg/kg significantly reduced the size of the tumor in two colon cancer mice models, orthotopic tumor allografts, and experimental metastatic models. This phytochemical has been revealed to induce apoptosis and inhibit inflammation and angiogenesis in these murine models of disease (Li et al., 2018). Wang B. et al. (2018) explored the potential anti-cancer effects of bigelovin on human liver cancer *in-vitro* and *in-vivo*. They reported that liver cancer cell lines, HepG2 and SMMC-7721, underwent apoptosis following bigelovin treatment, which activated PARP and caspases 3, and 9, augmented Bax, and suppressed Bcl-2 expressions. Bigelovin also induced autophagy in these cancer cell lies. Xenograft model of HepG2 tumor treated with bigelovin significantly suppressed in a dose-dependent manner. Zeng et al. (2009) reported anti-proliferative effects of bigelovin on human monoblastic leukemia U937 cells with an IC_50_ value of 0.47 µM. They identified morphological features of apoptosis in bigelovin-treated cells. The authors also demonstrated that bigelovin treatment increased the percentage of Annexin V positive U937 cells and caused the cell cycle arrest at the G0/G1 phase.

### 4.7 Britannin

Britannin is a sesquiterpene lactone isolated from *Inula aucheriana* with anti-cancer effects on different cancer cells (Moghadam et al., 2012). This sesquiterpene lactone has been observed to stimulate apoptosis in human pancreatic cancer cell lines AsPC-1 and Panc-1. Its apoptosis-inducing effects on these cell lines are mediated by the upregulation of BCL-2 and downregulation of BAX together with activating caspase 3, cytochrome C efflux from mitochondria into the cytosol, PARP cleavage, and ROS generation (Moeinifard et al., 2017). Mohammadlou and co-workers examined the anticancer effect of britannin on acute lymphoblastic leukemia (ALL) cell lines NALM-6, REH, and JURKAT cells. They reported that NALM-6 cells had the highest sensitivity to this sesquiterpene lactone. In this cell line, britannin induced ROS-dependent G1 cell cycle arrest (Mohammadlou et al., 2021). In another study, they assessed the cytotoxic effects of britannin alone or in combination with vincristine on another ALL cell line MOLT-4. They found that britannin hampered the proliferation of MOLT-4 cells and caused G1/S phase cell cycle arrest. Britannin pro-apoptotic effects on this cell line were exerted by a mechanism that involved ROS formation, Bax and Bim upregulation, caspase3 activation, Bcl-2 downregulation, and XIAP inhibition. Britannin also showed a synergistic effect with Vincristine on MOLT-4 cells (Mohammadlou et al., 2022b). Hamzeloo-Moghadam et al. (2015) investigated the anticancer activity of britannin on two human breast cancer cell lines MCF-7 and MDA-MB-468. Induction of the activity of caspases 3 and 9 following britannin treatment affirmed the pro-apoptotic effect of this phytochemical on breast cancer cells. The Western blot results indicated the inhibitory activity of britannin on anti-apoptotic Bcl-2 and pro-apoptotic Bax proteins. It also increased ROS formation, blocked MMP, and increased cytosolic levels of cytochrome C in MCF-7 and MDA-MB-468 breast cancer cells. Rajabi et al. (2023) indicated that the apoptosis-inducing effect of britannin on MCF-7 cells involves the inhibition of the JAK/STAT signaling pathway. Evaluating the effect of britannin on CML cell line K562 and acute myeloid leukemia (AML) cell line U937 by Mohammadlou et al. (2022a) revealed its anti-proliferative activity against both cell lines. However, the proliferation of K562 cells was suppressed at higher concentrations of britannin in comparison to U937 cells. They reported that britannin treatment significantly augmented the expression of pro-apoptotic genes BAX, BAD, and BID and remarkably suppressed the expression of anti-apoptotic genes BCL-2, BCL-XL, and MCL-1 in both cell lines. However, they have not measured the protein expression of these genes and the activated forms of caspase enzymes. A study by Cui Y. Q. et al. (2018) unraveled that britannin significantly induced apoptosis in human liver cancer cell line HepG2 in a ROS-dependent manner. Activation of three caspase enzymes 3, 8, and 9 due to britannin treatment of HepG2 cells indicated the involvement of both extrinsic and intrinsic apoptotic pathways. Moreover, they showed that britannin could stimulate autophagy in these cancer cells. A xenograft model of HepG2-derived tumor using BALB/c nude mice uncovered that britannin has a suppressing effect on tumor growth *in-vivo*. Zeng et al. (2020) evaluated the *in-vitro* and *in-vivo* effects of britannin on the growth of human prostate cancer cell lines (PC-3, PC-3-LUC, and DU-145). The *in-vitro* study showed the anti-proliferative and pro-apoptotic activity of britannin in all three cell lines. Mechanistic experiments on PC-3 cell lines revealed upregulated levels of pro-apoptotic protein Bax and downregulated levels of Bcl-2 protein in this cell line. The authors have claimed that an increase in caspase 9 and caspase 3 expression levels was observed in our preliminary pathway target study. However, there is no data regarding the western blotting of these two enzymes in that study. The results of their *in-vivo* experiment uncovered that britannin inhibited the growth of PC-3-LUC tumors.

### 4.8 Costunolide

Costunolide is a sesquiterpene lactone derived from *Saussurea lappa*. Tabata et al. (2015) reported anticancer effects of costunolide on four human neuroblastoma cell lines, including IMR-32, NB-39, SK-N-SH, and LA-N-1. They provided evidence to show that costunolide stimulated apoptotic characteristics in IMR-32 cells. The mechanistic study led to the observation of caspase 7 and PARP activation after 24 h treatment of IMR-32 cells with costunolide. However, this phytochemical compound did not affect the expression of caspase 3, caspase 9, Bax, and Bcl-2, suggesting that the mitochondrial pathway had no role in the apoptosis-inducing activity of costunolide. Choi et al. (2012) found that costunolide inhibits cell proliferation in MDA-MB-231 breast cancer cells. They also established that costunolide triggers apoptosis in this cell line by acting on the Fas receptor and activation of caspases 3 and 8 and PARP cleavage. However, it had no significant effect on MMP and expression of Bcl2 and Bax proteins. These data may suggest that costunolide induces apoptosis in MDA-MB-231 through the extrinsic pathway. Costunolide inhibited the proliferation of human esophageal cancer cells Eca-109 in a dose-dependent manner. It also decreased MMP and increased intracellular ROS levels to induce apoptosis in these cells. The apoptosis-inducing activity of costunolide was shown by increasing Bax, decreasing Bcl-2, and activating caspase 3 and PARP1 in the Eca-109 cell line (Hua et al., 2016a). Two studies have shown the anticancer effects of costunolide on three different gastric cancer cell lines. In one study, the effect and mechanism of costunolide on two gastric cancer cell lines HGC-27 and SNU-1 were assayed. The obtained data evidenced the anti-proliferative and pro-apoptotic activity of costunolide in both cell lines. Further experiments identified that costunolide upregulated Bax and Bak, downregulated Bcl-2, and activated caspase 3 and PARP1 in both cell lines. However, it had no effect on the levels of fas, DR4, fasL, and caspase 8, suggesting the involvement of the intrinsic apoptosis pathway in the apoptosis-inducing effects of costunolide on gastric cancer cells (Xu et al., 2021). In the other study, costunolide induced apoptosis in human gastric adenocarcinoma BGC-823 cells *in vitro* and *in vivo*. The results of that study revealed that costunolide hampered MMP together with an increase in the expression of Bax, cleaved caspase 9, cleaved caspase 7, cleaved caspase 3, and cleaved PARP proteins. Costunolide also lowered the expression of Bcl-2, pro-caspase 9, pro-caspase 7, pro-caspase 3, and PARP proteins. In addition, costunolide prohibited the growth of the xenograft model of BGC-823 cells in athymic nude mice. This sesquiterpene lactone similarly activated caspase 9 and caspase 3 along with the upregulation of Bax and downregulation of Bcl-2 proteins in xenografted tumors (Yan et al., 2019). Choi and Lee (2009) reported that costunolide induced apoptosis in human promonocytic leukemia U937 cells by increasing the production of ROS and suppression of Bcl-2. Treatment of the xenograft mice model of this cancer significantly suppressed tumor growth and increased survival of the animals. In human lung cancer A549 cells, costunolide activated caspases 3 and 9, augmented Bax, diminished Bcl-2, cleaved PARP, and increased cytosolic concentration of cytochrome C to induce apoptosis in these cancer cells in a ROS-dependent manner (Wang et al., 2016). In another human lung squamous carcinoma cell line SK-MES-1, costunolide treatment significantly triggered apoptosis and induced cell cycle arrest at G1/S transition. Costunolide induced apoptosis in these cells via augmenting the expression of Bax, suppressing Bcl-2, and activating caspase 3 and PARP enzymes (Hua et al., 2016b). Costunolide isolated from the stem of *Lycium shawii* initiated apoptosis in oral squamous cell carcinoma (OSCC) cell line CAL 27. This compound upregulated Bak, amplified the levels of cleaved caspases 3 and 6, and anti-apoptotic factor BCL2L1 in this cell line (Shams et al., 2023). In platinum-resistant human ovarian cancer cells (MPSC1 and SKOV3), costunolide has been shown to act stronger than cisplatin in decreasing cell viability. Costunolide promoted apoptosis of MPSC1 and SKOV3 cell lines through activating of caspases 3, 8, and 9 along with the suppression of Bcl-2 and production of ROS (Yang et al., 2011). The combination of costunolide and doxorubicin stimulated the induction of mitochondria-dependent apoptosis in two prostate cancer cell lines PC-3 and DU-145. This combination induced the cleavage of caspase 3, caspase 9, and PARP. It also has been shown to upregulate pro-apoptotic factors Bax and Bak and downregulated anti-apoptotic factors Bcl-2 and Bcl-xL in both cancer cell lines (Chen et al., 2017). This natural sesquiterpene compound decreased the viability of four human renal carcinoma cell lines (786-O, A-498, ANCH, and 769-P). Further experiments revealed that costunolide increases the ratio of Bax to Bcl-2, decreases MMP, and augments the release of cytochrome C into the cytoplasm. It also significantly activated caspase 9, caspase 3, and PARP enzyme, but had no effect on caspase 8 in 769-P cells. Other experiments showed that costunolide induces both apoptosis and autophagy in this cell line in a ROS-mediated way (Fu et al., 2020). Zhang et al. (2016) also observed similar data in their study on osteosarcoma. They demonstrated that costunolide had apoptotic activity in human osteosarcoma U2OS cells. Moreover, they found that costunolide increases the Bax/Bcl-2 ratio, promotes MMP loss, and induces cytochrome C release to trigger apoptosis in U2OS cells in a ROS-dependent manner. Their results also showed that costunolide increased cleaved forms of caspases 3 and 9 with no remarkable effect on the activation of caspase 8. Lee et al. (2021) uncovered the pro-apoptotic activity of costunolide in the A431 human epidermoid carcinoma cell line. They reported that costunolide induced A431 cell apoptosis by increasing the amount of cleaved caspase 3 and PARP. They further identified that costunolide induces apoptosis in these cancer cells by amplifying the expression of the pro-apoptotic protein Bax while downregulating anti-apoptotic proteins Bcl-2 and Bcl-xL.

### 4.9 Cynaropicrin

Cynaropicrin is a sesquiterpene lactone extracted from *Saussurea lappa* with cytotoxic effects on human leukemia cell lines, including U937, Eol-1, and Jurkat T cells. It has been reported that cynaropicrin induces apoptosis and cell cycle arrest at the G1/S phase in U937 cells in a ROS-mediated manner (Cho et al., 2004). Cynaropicrin has been described to cause apoptotic cell death in the human glioblastoma U-87 MG cell line. Cynaropicrin exerts this effect on U-87 MG through inducing intracellular ROS generation, reducing MM, and promoting the release of cytochrome C into the cytoplasm of these cancer cells. It also led to the activation of caspase 3 and caspase 9, suggesting the involvement of the mitochondrial pathway of apoptosis in cynaropicrin effects on U-87 MG cells (Rotondo et al., 2022). Liu T. et al. (2019) conducted a study to evaluate the effect of cynaropicrin on the Hela human cervical cancer cell line. They reported that cynaropicrin induces apoptosis in Hela cells by a novel mechanism that involves the thioredoxin system. They also asserted that this sesquiterpene functions by activating caspase 3 and inducing the production of ROS to trigger apoptosis in Hela cells. However, they have not assessed other apoptosis markers to affirm their obtained data. Yang R. et al. (2022) studied the effect of cynaropicrin on neuroblastoma models *in-vitro* and *in-vivo*. *In-vitro*, two SK-N-BE (2) and SH-SY5Y cell lines were shown to undergo apoptotic cell death by a mechanism that increases the ratio of Bax/Bcl-2 and leads to the activation of caspase 3 and PARP enzymes. Their *in-vivo* results evidenced the growth-inhibiting effect of cynaropicrin on a xenograft model of neuroblastoma in nude mice.

### 4.10 Dehydrocostus lactone

Dehydrocostus lactone (DHL) is a natural sesquiterpene lactone isolated from *Saussurea lappa* root with a variety of biological activities. Gu et al. (2023) reported the anticancer effect of DHL on HepG2 human hepatocellular carcinoma cells with an IC_50_ value of 20.33 µM. They also performed a TUNEL assay and the results showed that DHL is a pro-apoptotic agent against HepG2 cells. However, they did not evaluate any of the apoptosis markers to affirm their data. Peng et al. (2017) conducted a study to examine the effects of DHL, costunolide, their combination and volatile oil from saussurea lappa root (VOSL) on two human breast cancer cell lines MCF-7 and MDA-MB-231 cell *in-vitro* and *in-vivo*. The results showed that all compounds had anti-proliferative and pro-apoptotic effects on the two cell lines. Besides, all compounds significantly increased the expression of Bax and decreased the expression of Bcl-2. The compounds also decreased the levels of p-BID. These data reveal the implication of the mitochondrial pathway of apoptosis. In another study by Kuo et al. (2009) on MCF-7 and MDA-MB-231 cell lines, it was illustrated that DHL induces upregulation of pro-apoptotic Bax and Bad proteins and promotes downregulation of anti-apoptotic factors Bcl-2 and Bcl-XL along with the activation of caspase 9. DHL also stimulated the translocation of apoptosis-inducing factor (AIF) and endonuclease G (EndoG) from mitochondria into and nucleus in both breast cancer cell lines. Jiang et al. (2015) reported that DHL induces apoptosis in two cervical cancer cell lines Hela and C33a at doses of 2.5, 5, and 10 μg/mL. However, they did not check any of the apoptosis markers. Cai et al. (2019) described the anti-viability and apoptosis-inducing effect of DHL on human chronic myeloid leukemia (CML) K562 cells. However, they only measured the expression of Bcl-2 and Bax. The results evidenced that DHL upregulated Bax and downregulated Bcl-2. In another study on the same cell line, they reported that DHL triggers apoptosis by a mechanism that involves the suppression of different cyclin molecules and three anti-apoptotic factors, including Bcl-2, Bcl-xL, and Mcl-1. Also, DHL increased Bax level, ROS production, and disrupted MMP to induce apoptosis in these cancer cells (Cai et al., 2017). Long et al. (2019) explored the antiproliferative properties of DHL in human gastrinoma cancer cell line BON-1. They provided limited data to show that DHL had apoptotic activity against BON-1 cancer cells. They reported IC_50_ values of 71.9 μM and 52.3 μM at 24 and 48 h DHL treatment and observed sub-G1 cell cycle arrest and MMP loss in BON-1 cells. Peng and co-workers studied the pro-apoptotic effects of DHL on Eca109 and KYSE150 esophageal cancer cells. They found that the apoptosis-stimulating effect of DHL on these cells involves the upregulation of Bcl-2, downregulation of Bax, and activation of caspases 3 and 9, and PARP enzymes in a ROS-dependent manner. In addition, DHL suppressed the growth of Eca109 tumor xenograft in nude mice by similar changes in above mentioned markers in tumor tissue (Peng et al., 2022). In human glioblastoma cell line U87, DHL significantly inhibited in viability and promoted apoptosis. This sesquiterpene significantly elevated the expression of Bax and the levels of cleaved forms of caspases 3 and 9. It also triggered the release of cytochrome C into the cytoplasm of U87 cells and suppressed the expression of the Bcl-2 protein (Wang et al., 2017). DHL inhibits the growth of laryngeal carcinoma *in-vitro* and *in-vivo*. Zhang and co-workers provided evidence to prove that DHL could induce apoptosis in laryngeal cancer cells Hep-2 and TU212 cells by inducing the expression of Bax and activity of caspase 3, caspase 9, and PARP. DHL treatment of the Hep-2 nude mouse xenograft model also led to the same results in addition to significant suppression of tumor growth (Zhang et al., 2020). Two hepatocellular carcinoma (HCC) cell lines, including HepG2 and SK-HEP-1, treated with DHL underwent apoptosis, which was identified by increased DNA damage and G1-phase cell cycle arrest. The mechanism of DHL in promoting apoptosis in these cells involved the upregulation of Bax and downregulation of Bcl-2 in addition to PARP activation. HepG2 xenograft in mice was significantly decreased in size due to DHL treatment (Tian et al., 2023). Hsu Y. L. et al. (2009) found that DHL inhibited the proliferation and induced apoptosis of HepG2 and PLC/PRF/5 cells. DHE acted by upregulating pro-apoptotic factors Bax and Bak, downregulating anti-apoptotic factors Bcl-2 and Bcl-XL, and translocation of AIF and Endo G to the nucleus. Hung et al. (2010) investigated the anticancer effect of DHL on human non-small cell lung cancer cell lines, A549, NCI-H460, and NCI-H520. Their results illustrated that DHL decreased the proliferation of all three cell lines but induced apoptosis in A549 and NCI-H460 cells. They also measured a variety of genes and proteins but no apoptosis marker was assessed in that study. Hsu H.-F. et al. (2009) observed that DHL suppresses the proliferation of human lung cancer A549 cells. Their further experiments demonstrated that the A549 cells underwent cell cycle arrest at the sub-G1 phase by a mechanism that activated caspases 3 and 9 with no effect on caspase 8. An *in-vitro* study by Sheng et al. (2018) showed that DHL diminished the proliferation of lung cancer cells (A549 and H460) and exerted synergistic effects on antiproliferative activity of doxorubicin. DHL induced apoptosis in lung cancer cells by activating caspase 3, caspase 9, and PARP together with downregulation of anti-apoptotic factor Bcl-2. Choi and Ahn designed a study to examine the pro-apoptotic activity of DHL on human ovarian cancer SK-OV-3 cells. The results showed that DHL treatment led to a significant cell cycle arrest at the G2/M phase through activating caspase 3 and PARP enzymes, overexpression of Bax, underexpression of Bcl-2, and release of cytochrome C into the cytoplasm of SK-OV-3 cells (Choi and Ahn, 2009). DHL has been shown to trigger apoptosis in human prostate cancer DU145 cells via elevating the levels of cleaved caspases 8, 9, 7, and 3 and PARP. It also amplified the levels of pro-apoptotic proteins Bax, Bak, Bok, Bik, truncated Bid (t-Bid), and Bmf. DHL increased cytosolic levels of cytochrome C and decreased Bcl-xL expression in DU145 cells (Kim et al., 2008). Kretschmer et al. (2012) conducted a study to explore the anti-human soft tissue sarcoma effect of DHL on SW-872, SW-982, and TE-671 cell lines. They found that DHL leads to a significant decrease in the number of cells in the G1 phase and an increase of cells in the S and G2/M phases. Further, enhanced activity of caspase 3/7, and increased levels of cleaved caspase-3 and cleaved PARP were observed in DHL-treated cell lines.

### 4.11 Deoxyelephantopin

Deoxyelephantopin is a sesquiterpene lactone isolated from *Elephantopus scaber*, which is a Chinese medicinal plant with anti-inflammatory and anticancer properties. Mehmood et al. (2017b) explored the anti-proliferation and apoptosis-stimulating effect of deoxyelephantopin on HepG2 cells. They found that deoxyelephantopin-mediated apoptosis was accompanied by the formation of ROS, redox imbalance, MMP loss, Bcl-2 underexpression, Bax overexpression, cytochrome C release, caspases 3 activation, and PARP cleavage HepG2 cells. Huang et al. (2010) conducted a study to assess the anti-breast cancer effect of deoxyelephantopin on a highly metastatic mouse breast adenocarcinoma cell line TS/A and a TS/A tumor model in BALB/c mice. They found that deoxyelephantopin prohibited cell proliferation, migration and invasion of TS/A cells and induced G (2)/M arrest and apoptosis in TS/A cells. Regarding the apoptosis-inducing effect of deoxyelephantopin, they observed the decreased levels of initiator procaspases 8 and 9 and executioner procaspases 3, 7, and 6 together with the activation of PARP enzyme. *In-vivo*, they showed that pre-treatment of mice with deoxyelephantopin is more effective than post-treatment in suppressing the growth of the metastatic TS/A cells. Deoxyelephantopin has antiproliferative and apoptosis-promoting effects on human cervical squamous cell carcinoma cell line SiHa. Its effect on apoptotic factors involves the activation of caspases 3, 7, 8, and 9, upregulation of Bax, and downregulation of Bcl-2 and Bcl-xL in a ROS-dependent mechanism (Farha et al., 2014). In another human cervical cancer cell line HeLa, deoxyelephantopin was reported to trigger apoptosis and cell cycle arrest at the G2/M phase characterized by the activation of three apoptosis-associated enzymes, including caspase 3, caspase 9, and PARP (Zou et al., 2008). Deoxyelephantopin has been shown to induce apoptosis in colon cancer cell lines (HCT116 and sw620) and the HCT116 xenograft mice model of the disease. *In-vitro*, deoxyelephantopin treatment led to G2/M phase arrest and subsequent apoptosis by acting on the suppression of Bcl-2 and activation of caspase 3 and PARP. *In-vivo*, it caused a significant reduction in the tumor volume of diseased mice (Ji et al., 2022). In another study on HCT116 cells, deoxyelephantopin caused morphological changes associated with apoptotic cell death in this cancer cell line. This sesquiterpene significantly led to the activation of caspase 3 and PARP cleavage to trigger apoptosis in HCT116 cells (Chan et al., 2016). Su and co-workers identified that deoxyelephantopin treatment of the human nasopharyngeal cancer cell line resulted in cell cycle arrest in the S and G2/M phases. Western blotting analysis uncovered that deoxyelephantopin-treated cells had elevated levels of pro-apoptotic Bax, Bad, Bok, Bmf, and PUMA proteins as well as a significant release of cytochrome C into the cytoplasm of CNE cells. It also suppressed the expression of Bcl-2 and Bcl-xL proteins with a significant decrease in MMP and a remarkable activation of caspases 3, 7, 8, 9, and 10 (Su et al., 2011). The exposure of MG-63 and U2OS human osteosarcoma cell lines to deoxyelephantopin has been revealed to increase the intracellular ROS levels, Bax/Bcl-2 ratio, cleaved caspase 3, cleaved caspase 9, and cleaved PARP leading to the initiation of apoptotic cell death in both cancer cell lines (Zou et al., 2017). Ji et al. (2020) evaluated the effect of deoxyelephantopin on pancreatic cancer *in-vitro* and *in-vivo*. *In-vitro*, two human pancreatic cancer cell lines BxPC-3 and CFPAC-1 were treated with different doses of deoxyelephantopin and the results showed that the expression of Bax, cytoplasmic levels of cytochrome C, and the levels of cleaved forms of caspases 3 and 9 were significantly augmented in both cancer cell lines in comparison to untreated cell lines. The expression of anti-apoptotic protein Bcl-2 was decreased in BxPC-3 and CFPAC-1 cell lines. *In-vivo*, treatment of the BxPC-3 cell xenograft model of pancreatic cancer with deoxyelephantopin led to a significant decrease in tumor size.

### 4.12 Ergolide

Ergolide is a sesquiterpene lactone derived from *Inula Brittanica* with promising anti-proliferative activity in some cancers such as metastatic uveal melanoma (Sundaramurthi et al., 2023). Two studies have evaluated the anti-proliferative and apoptotic activity of ergolide on cancer cells. In one study, treatment of ALL cell lines (Nalm6 and MOLT-4) with ergolide has resulted in the induction of apoptosis in a ROS-mediated way, leading to the activation of caspase 3 and PAPR enzymes. Furthermore, ergolide was found to upregulate proapoptotic factors Bax and Bim and downregulate anti-apoptotic factors Bcl-2 and XIAP. The increase in the ratio of Bax to Bcl-2 in both Nalm6 and MOLT-4 cell lines (Song et al., 2005). In the second study, Jurkat T cells were treated with different concentrations of ergolide to assess the anti-ALL effect of this agent *in-vitro*. Ergolide-induced apoptosis in Jurkat T cells was observed by DNA fragmentation, caspase 3 activation, and PARP cleavage. Moreover, ergolide treatment resulted in the upregulation of Bax, downregulation of Bcl-2 and XIAP, leading to the release of cytochrome C into the cytoplasm of Jurkat T cells (Song et al., 2005).

### 4.13 Eupalinolide

Eupalinolide J is a sesquiterpene lactone present in *Eupatorium lindleyanum* DC. We, et al. studied the anticancer effect of eupalinolide J on two human prostate cancer cell lines PC-3 and DU-145. Their analyses showed that this sesquiterpene induced cell cycle arrest in both cell lines at the G0/G1 phase. It also disrupted MMP and upregulated the levels of cleaved forms of caspases 3 and 9 in PC-3 and DU-145 cell lines (Wu et al., 2020). Zhao et al. (2022) conducted a study to investigate the effect of eupalinolide O on triple-negative breast cancer (TNBC) *in-vitro* and *in-vivo*. They reported that eupalinolide O suppressed the proliferation and induced apoptosis of two TNBC cell lines MDA-MB-231 and MDA-MB-453. The mechanism of apoptosis induction by eupalinolide O implicated the disruption in MMP, increased formation of intracellular ROS, and elevated activity of caspase 3. This sesquiterpene also increased the expression of Bax, PARP, and caspase 9 and decreased the expression of Bcl-2 genes. *In-vivo*, tumor xenograft of both MDA-MB-231 and MDA-MB-453 cell lines demonstrated the growth inhibitory effect of eupalinolide O on both TNBC tumors with an increased level of Ki67 protein and intra-tumor ROS and decreased expression of caspase 3. Yang et al. (2016) evaluated the effect of eupalinolide O on another human MDA-MB-468 TNBC cell line *in-vitro*. They showed that eupalinolide O treatment resulted in cell cycle arrest in the G2/M phase. The expression of pro-apoptotic proteins Bax and Bad was significantly increased and the expression of anti-apoptotic proteins Bcl-2 and Bcl-xL was decreased in MDA-MB-468. eupalinolide O also increased the activated forms of caspase 3, caspase 9, and PARP but had no effect on caspase 8 activity in these cancer cells. Zhang et al. (2022) indicated that eupalinolide A could suppress the proliferation and growth of hepatocellular carcinoma cells and tumors. Eupalinolide A induced cell cycle arrest in tow hepatocellular carcinoma cell lines (MHCC97-L and HCCLM3) at the G1 phase but had no remarkable effects on apoptosis and autophagy markers. *In-vivo*, eupalinolide A significantly decreased the tumor volume of the xenograft models of MHCC97-L and HCCLM3 cell lines.

### 4.14 Gaillardin

As a novel sesquiterpene lactone isolated from aerial parts of *Inula oculus-christi*, gaillardin has recently been reported to have anticancer properties. It can induce apoptosis in breast cancer. Apoptosis-induction in these cancer cells is accompanied by disrupting MMP, increasing intracellular RO, and activating caspases 3, 6, and 9. It also is able to upregulate the pro-apoptotic protein Bax and downregulate the anti-apoptotic protein Bcl-2 in MCF-7 and MDA-MB-468 cell lines (Fallahian et al., 2015). Karami et al. (2020) identified that treatment of ALL cell lines (NALM-6 and MOLT-4) with gaillardin resulted in the cell cycle arrest at G0/G1 phase in a dose-dependent manner. Furthermore, they reported that gaillardin treatment induces apoptotic cell death both in NALM-6 and MOLT-4 cell lines by a mechanism that leads to the upregulation of the mRNA expression of caspase 3, Bax, and Bcl-2. Sayyadi et al. (2021) studied the anti-proliferative effect of gaillardin on the acute promyelocytic leukemia (APL) cell line, NB4. Flow cytometry data showed the apoptosis-stimulating effect of gaillardin on NB4 cells by decreasing the expression levels of the Bcl-2 gene and increasing mRNA levels of Bax and Bad. In gastric cancer cell lines (AGS and MKN45), gaillardin treatment significantly decreased cell viability and induced apoptosis in a time and concentration-dependent manner. Apoptosis induction in these cancer cell lines was shown by Bax and caspase 3 genes upregulation and Bcl-2 gene downregulation (Roozbehani et al., 2021).

### 4.15 Helenalin

Helenalin is a sesquiterpene lactone found in *Arnica montana* and *Arnica chamissonis* ssp. *Foliosa* with the capacity to induce apoptosis in Caki and ACHN renal cell carcinoma cells and human colon carcinoma HT29 and HCT116 cells (Jang et al., 2013). Helenalin has been evidenced to trigger cell death apoptosis-resistant Bcl-2 overexpressing cancer cells such as leukemic Jurkat, breast MCF-7, and pancreatic L6.3pl cell lines. In these cells, helenalin directly acts on Bcl-2 in the intrinsic pathway and induces ROS to trigger apoptosis in Jurkat cells with no significant effect on cytochrome C release, MMP, caspases, AIF, Omi/HtrA2, Apaf/apoptosome (Hoffmann et al., 2011). Dirsch et al. (2001) showed that helenalin induced apoptosis in leukemia Jurkat T cells which are CD95 death receptor-deficient and overexpress Bcl-2. They revealed that helenalin led to a time-dependent proteolytic activation of procaspase 3 and 8 in these cancer cells. Helenalin treatment also resulted in the release of cytochrome C and loss of MMP. Their data suggested helenalin as a novel agent to combat apoptosis resistance in Bcl-2-overexpressing cells.

### 4.16 Isoalantolactone

Isoalantolactone (IAL) is a sesquiterpene lactone naturally found in the roots of *Inula helenium* L. with cytotoxicity against different cancer cells. Li et al. (2022) observed that IAL induces G0/G1 phase cell cycle arrest, apoptosis, and autophagy in colorectal cancer (CRC) cell lines, including HCT116 and SW620. Pro-apoptotic activity of IAL in these cancer cell lines was shown by downregulation of anti-apoptotic proteins Bcl-2, Bcl-xL, and Mcl-1 as well as downregulation of pro-apoptotic protein Bax and activation of PARP enzyme in both cancer cell lines. *In-vivo* results evidenced that IAL suppressed HCT116 tumor growth in BALB/c-nu/nu mice by decreasing the protein expression of Bcl-2 and Bcl-xL and cleavage of PARP enzyme in tumor tissue. IAL has shown a significant cytotoxic effect on human gastric adenocarcinoma SGC-7901 cells. This sesquiterpene induced G2/M and S phase arrest and triggered apoptosis in these cancer cells which was associated with the downregulation of Bcl-2 and upregulation of Bax that led to the loss of MMP and activation of caspase 3 in SGC-7901 cells (Rasul et al., 2013b). Treatment of U87 human glioblastoma cell line with IAL has led to a significant increase in the level of Bax to Bcl-2 proteins that resulted in the release of cytochrome C into the cytoplasm of U87 cells and cleavage of caspases 3 and 9 and PARP. The xenograft model of BALB/c nu/nu mice using the U87 cell line was developed to evaluate the *in-vivo* effect of IAL. The results showed that IAL could significantly hamper tumor growth (Xing et al., 2019). The exposure of head and neck squamous cell carcinoma cell line (UM-SCC-10A) to IAL was associated with decreased proliferation and induced apoptosis. Apoptosis induction was associated with cell cycle arrest at the G1 phase, increased expression of Bax, decreased level of Bcl-2, mitochondrial release of cytochrome C, disruption of MMP, and cleavage of caspase 3 in UM-SCC-10A cells (Wu et al., 2013). It has been reported that IAL induced apoptosis in human erythroleukemia drug-resistant cell line K562/A02 through a mechanism that acts by increasing the level of intracellular ROS, augmenting the protein levels of Bcl-2, diminishing the protein expression of Bax, inducing cytochrome C release, and cleavage of caspase 3, caspase 9, and PARP (Cai et al., 2014). Kim et al. (2021) declared that treatment of human hepatocellular carcinoma Hep3B cells with IAL was accompanied by the induction of apoptosis. IAL upregulated death receptors such as DR4, DR5, and Fas, transformed Bid to t-Bid, increased the ratio of Bax to Bcl-2, induced cytochrome C release, induced the cleavage of caspases 8 and 9, and finally caused the activation of caspase 3 and PARP in Hep3B cells in a ROS-mediated apoptosis. These data show that IAL activated both pathways of apoptosis in hepatocellular carcinoma cells. IAL-treated lung squamous carcinoma SK-MES-1 cells have been found to undergo apoptotic cell death in a concentration-dependent manner. Downregulation of Bcl-2, upregulation of Bax, disruption of MMP, generation of ROS, activation of caspase 3, and cleavage of were the main alterations that were observed in IAL-treated SK-MES-1 cells (Jin et al., 2017). Di et al. (2014) investigated the effect of IAL on human osteosarcoma cell lines U2OS, MG-63, and Saos-2 cells. Apoptosis study on U2OS cells provided evidence to demonstrate that IAL may stimulate S and G2/M cell cycle arrest in a ROS-dependent way. It also decreased MMP, upregulated DR5, FADD and cleaved caspase 8. This agent promoted apoptosis in U2OS cells by suppressing Bcl-2, inducing Bax, and activating caspase 3 and its downstream substrate, PARP. Ovarian cancer cell lines (SKOV-3 and OVCAR-3) treated with IAL underwent cell cycle arrest at G2/M phase arrest and apoptosis. IAL exerted its apoptotic effects on these cancer cell lines by increasing intracellular ROS, amplifying Bax, suppressing Bcl-2, and activating caspase 3 and PARP (Xie et al., 2023). Two studies have been conducted to assess the effects of IAL on the proliferation and apoptosis in pancreatic cancer. In one study, it was found that IAL inhibited proliferation and triggered apoptosis in pancreatic cancer PANC-1 cells. Further analyses demonstrated that induction of apoptosis was associated with amplified levels of ROS, dissipated MMP, increased levels of cytosolic cytochrome C, increased expression of Bax, elevated active caspase 3, and decreased expression of Bcl-2 in a dose-dependent manner (Khan et al., 2012a). In the other study, IAL inhibited the proliferation of three pancreatic adenocarcinoma cell lines pancreatic carcinoma cell lines (PANC-1, AsPC-1, BxPC-3) while the apoptosis assay and its markers were evaluated in the PANC-1 cell line. The results indicated the induction of apoptosis PANC-1 cells by increasing cytosolic levels of caspase3 and Bax expression. No other apoptotic marker was measured in that study. Finally, the xenograft model of the disease in BALB/c nude male mice using all three cell lines showed that IAL could significantly suppress the growth of pancreatic adenocarcinoma tumors *in-vivo* (Zhang C. et al., 2021). A study on the effect of IAL on the induction of apoptosis in prostate cancer cells, including PC-3 and DU145, showed the amplification of the number of apoptotic cells in IAL-treated cell lines. Furthermore, IAL treatment increased the activity of caspases 3 and 9 and downregulated the expression of Bcl-2 protein in a dose-dependent manner. However, it is not clear these factors were changed in which type of these cancer cell lines (Chen W. et al., 2018). In another study, IAL induced apoptosis in prostate cancer PC3 cells through a mechanism that led to the generation of ROS, dissipation of MMP, upregulation of Bax, downregulation of Bcl-2 and survivin and activation of caspase 3 (Rasul et al., 2013a).

### 4.17 Isoalantolactone

Isoalantolactone, as a sesquiterpene lactone, is a natural compound found in the roots of *Inula helenium*. Only one study was found to evaluate the anti-cancer and pro-apoptotic effects of isocostunolide. In that study, it was found that isocostunolide could significantly exert cytotoxic effects on three different cancer cell lines (melanoma, A2058; colorectal, HT-29; and hepatocarcinoma, HepG2). Flow cytometry data showed that isocostunolide effectively induced apoptosis in A2058 cancer cells by a significant loss of G0/G1 phase cells. Further analyses revealed that apoptotic activity of isocostunolide in A2058 cells was associated with the activation of caspases 3 and 8, truncation of Bid, amplification of Fas, cleavage of PARP, downregulation of Bcl-2, and release of cytochrome C into the cytosol of A2058 cells (Chen et al., 2007).

### 4.18 Isodeoxyelephantopin

Isodeoxyelephantopin is a sesquiterpene lactone derived from *Elephantopus scaber* Linn. Isodeoxyelephantopin has been shown to act as an apoptotic agent against the human leukemia KBM-5 cell line. Ichikawa et al. (2006) reported that isodeoxyelephantopin triggers apoptosis in KBM-5 cells by blocking the expression of tumor necrosis factor-α (TNF-α)-induced anti-apoptotic factors such as IAP1/2, Bcl-2, Bcl-xL, Bfl-1/A1, TRAF1, FLIP, and survivin. Verma et al. (2019) evaluated the pro-apoptotic activity of isodeoxyelephantopin against breast cancer MDA-MB-231 and MCF-7 cell lines and underlying mechanisms. They reported that isodeoxyelephantopin suppressed the proliferation of both MDA-MB-231 and MCF-7 breast cancer cell lines and induced an accumulation of cells in the sub-G1 and G2/M phases. Isodeoxyelephantopin treatment of MDA-MB-231 cells led to the MMP loss, and cleavage of caspases 7 and 9, and PARP as well as a significant suppression in the protein level of anti-apoptotic factors Bcl-2 and Bcl-xL. Isodeoxyelephantopin antiproliferative effects on breast carcinoma T47D cells and lung carcinoma A549 cells were demonstrated. They declared that isodeoxyelephantopin suppresses the proliferation of A549 and T47D cells in a dose- and time-dependent manner. They also identified that isodeoxyelephantopin–treated cells die by apoptotic mechanism, which was associated with increased activity of caspase 3 and cell cycle arrest at the G2/M phase (Kabeer et al., 2014).

### 4.19 Janerin

As a sesquiterpene lactone isolated from *Centaurothamnus maximus,* janerin is a cytotoxic natural compound. It has been reported that janerin could reduce the proliferation of THP-1 AML cell line in a concentration-dependent way. Janerin treatment of these cancer cells has been shown to induce cell cycle arrest at the G2/M phase and apoptosis. Western blotting analysis illustrated the upregulation of pro-apoptotic factor Bax, activation of PARP and caspase 3, and the downregulation of the anti-apoptotic marker Bcl-2 in THP-1 cells (Ahmed et al., 2021).

### 4.20 Lactucopicrin

As a sesquiterpene lactone derived from the plant *Lactuca virosa*, Lactucopicrin has anticancer activity against different cancer types. In a study by Meng et al. (2019), the anti-viability and pro-apoptotic effect of lactucopicrin was assessed on human osteosarcoma Saos-2 cells. As an apoptosis-inducing agent, lactucopicrin induced cell cycle arrest in Saos-2 cells at the sub-G1 phase, upregulated the expression of Bax, and downregulated the level of Bcl-2 proteins. Zhang X. et al. (2018) evaluated the anticancer effects of lactucopicrin on human skin cancer SKMEL-5 cells. The results evidenced significant anti-proliferative and pro-apoptotic effects of this sesquiterpene on the SKMEL-5 cells. They implied that the anticancer effects of this agent were due to the induction of apoptosis, which was confirmed by the increased expression of Bax and decreased expression of Bcl-2 proteins in SKMEL-5 cells. In a study by Rotondo et al. (2020), the anticancer effects of lactucopicrin on glioblastoma continuous cell line U87Mg were examined. They reported a dose and time-dependent reduction of U87Mg cell proliferation and a cell cycle arrest at the G2/M phase in a ROS-mediated manner following lactucopicrin treatment. Their further analyses clarified that lactucopicrin stimulated apoptosis by downregulating procaspase 6 and activating PARP. However, no other apoptosis markers were evaluated in that study.

### 4.21 Parthenolide

Parthenolide is a sesquiterpene lactone isolated from *Tanacetum parthenium* that has anticancer effects on some types of cancer (Guzman et al., 2005). Guzman et al. (2005) reported apoptosis-stimulating activity of parthenolide against AML cells in a ROS-dependent way. However, they did not evaluate apoptosis biomarkers. Jorge et al. (2023) reported that parthenolide induced apoptotic cell death in several lymphoid malignancies, including NCI-H929 (MM), Farage (GCB-DLBCL), Raji (BL), 697 and KOPN-8 (B-ALL), and CEM and MOLT-4 (T-ALL) cell lines. The results showed cell cycle arrest, MMP decline, ROS production, activated caspase 3, and increased Fas-L in parthenolide-treated cell lines. An *in-vitro* study conducted by Cheng and Xie (2011) showed that parthenolide has the ability to induce apoptosis in human bladder cancer cells via reducing the protein expression of Bcl-2 and activating the PARP enzyme. Treatment of 5637 cells with parthenolide led to G1 phase cell cycle arrest. They did not check other apoptosis markers to affirm their obtained data. In an *in-vitro* study, Sweeney and co-workers identified that parthenolide had pro-apoptotic effects on the breast cancer MDA-MB-231 cell line. Further, *in-vivo* experiments showed non-significant enhanced survival in animal models (Sweeney et al., 2005). No other apoptosis biomarker was measured in that study. Berdan et al. (2019) showed anti-proliferative activity of parthenolide evidenced by propidium iodide-positive and annexin-V-positive breast cancer cells, 231MFP and HCC38. Given that parthenolide treatment led to the activation of caspase-3/7, they suggested that a portion of the cell death is apoptotic. However, no other apoptosis markers were estimated in that study. Ghorbani-Abdi-Saedabad et al. (2020) conducted a study to estimate the apoptosis-stimulating effect of parthenolide on breast cancer cell line MDA-MB-468. The results of real-time PCR indicated that the parthenolide-treated cells showed upregulated levels of Bax and downregulated levels of Bcl2 genes. The authors have asserted that parthenolide treatment resulted in a significant increase in caspase 3 protein level. However, there is no Western blotting image in the paper. In a study by Al-Fatlawi et al. (2015), the effect of parthenolide on the proliferation and apoptosis of human cervical cancer (SiHa) and breast cancer (MCF-7) cell lines were evaluated. According to their results, parthenolide suppressed the proliferation of SiHa and MCF-7 cell lines. Moreover, parthenolide-treated cells had upregulated levels of p53, Bax, caspases 3, 6, and -9 genes and downregulated mRNA of the Bcl-2 gene. Parthenolide effects on apoptosis Hela cervical cancer cells were also evaluated in a study by Jeyamohan et al. (2016). Parthenolide treatment decreased HeLa cell viability and induced mitochondrial-mediated apoptosis by activation of caspase 3, upregulation of Bax, and downregulation of Bcl-2. Duan et al. (2016) reported that parthenolide induces ROS-mediated apoptosis in HeLa cells but no marker of apoptosis was measured. Cholangiocarcinoma cell lines (SCK, JCK, Cho-CK, and Choi-CK) have been shown to undergo apoptosis due to parthenolide treatment in an investigation by Kim et al. (2005). They reported that parthenolide downregulated the Fas mRNA expression in all cell lines and decreased the FasL gene expression in the three cell lines but not in the SCK cells. Moreover, Parthenolide showed no effect on the expression of the Bcl-2 and Bcl-xL proteins in all cell lines. Although parthenolide had no effect on Bad expression, cleavage of Bid and upregulation of Bak and Bax were observed in all four cell lines. Flores-Lopez et al. (2018) suggested that parthenolide may induce apoptosis in CML cell lines (K562, KCL-22, and Meg-01) compared with untreated but did not measure apoptosis-related markers to confirm their observations. In colorectal cancer cells, parthenolide exposure could lead to apoptotic cell death of SW620 cells evidenced by the inhibited expression of anti-apoptotic proteins (Bcl-2 and Bcl-xL) and activated caspase 3 enzyme (Liu Y. C. et al., 2017). Kim et al. (2012) conducted *in-vitro* and *in-vivo* experiments to unravel parthenolide-mediated cell death in human colorectal cancer cell lines HT-29, SW620, and LS174T. They demonstrated the apoptosis-inducing activity of parthenolide in these cancer cells using Annexin V assay and Hoechst 33,258 staining. However, apoptosis markers were measured in HT-29 cells, which showed mitochondrial-mediated apoptosis evidenced by downregulation of Bcl-2 and upregulation of Bax and t-Bid, leading to the release of cytochrome C and caspase activation. Intraperitoneal injection of parthenolide revealed significant suppression of tumor growth and angiogenesis in the xenograft model. Anderson and Bejcek reported limited data on the pro-apoptotic effect of parthenolide on glioblastoma U-87 MG cells confirmed by the activation of caspases3/7 (Anderson and Bejcek, 2008). Hepatocellular carcinoma cell lines, including HepG2, MHCC 97H, and Huh7 treated with parthenolide triggered mitochondria-dependent apoptosis, which was affirmed by the upregulation of Bax, downregulation of Bcl-2 and cleavage of caspase 3. *In-vivo* model of the disease using the H22 cell line showed that parthenolide was not effective in suppressing tumor growth but the combination of parthenolide with arsenic trioxide led to significant tumor suppression (Yi et al., 2022). Parthenolide treatment of human lung cancer A549 cells has been uncovered to activate caspase 3 and caspase 9 in a way that involves the NF-kappaB signaling pathway (Fang et al., 2010). In an *in-vitro* experiment, it was proven that parthenolide induced apoptotic cell death in A375 melanoma cells. The cells exposed to parthenolide had decreased MMP, increased levels of ROS, and augmented the activity of caspase 3 (Lesiak et al., 2010). Ovarian cancer cell lines OVCAR-3 and SK-OV-3 were treated with parthenolide in a study by Kwak, et al. The results suggested that parthenolide treatment could decrease cytosolic Bid, Bcl-2, Bcl-xL, and survivin levels in addition to a significant increase in a cytochrome C release, and Bax levels, and cleaved PARP-1 OVCAR-3 cells. Further, this sesquiterpene may enhance the activities of caspases 3, 8, and 9 in OVCAR-3 and that of caspase 3 in SK-OV-3 cells in a dose-dependent way (Kwak et al., 2014). Parthenolide has been shown to trigger both apoptosis and autophagy in human pancreatic cancer Panc-1 and BxPC3 cells. To induce apoptosis in these cancer cells, parthenolide induced the cleavage and activation of caspase 3 and PARP enzymes (Liu W. et al., 2017).

### 4.22 Santamarine

Santamarine is a sesquiterpene lactone found in *Magnolia grandiflora* and *Ambrosia confertiflora* plants. It has been shown to have potential anticancer activity against HepG2 cells. This sesquiterpene can inhibit the proliferation and induce apoptosis of these cancer cells. Induction of apoptosis has been found to be accompanied by the generation of ROS, MMP loss, and elevation of the cellular levels of Bax, Bad, and cytochrome C. It also triggers the activation of caspases 3, 8, and 9 and the cleavage of Bid and PARP enzymes in the HepG2 cell line (Mehmood et al., 2017a). In A549 lung adenocarcinoma cells, santamarine promotes apoptosis by the induction of oxidative stress. The induction of apoptosis in this cell line has been shown by downregulation of Bcl-2, upregulation of Bax, disruption of MMP, activation of caspase 3, and cleavage of PARP in a dose-dependent manner (Wu et al., 2017). The other study on cervical cancer cell line HeLa showed that santamarine treatment induces apoptotic cell death in these cancer cells by a mechanism that involves the accumulation of high levels of ROS and activation of caspase 3. No other markers of apoptosis were evaluated in that study (Zhang J. et al., 2021).

### 4.23 Scabertopin

Scabertopin is one of the major sesquiterpene lactones derived from *Elephantopus scaber* L. We found only one study on the apoptosis-inducing effects of scabertopin on cancer cells. Gao et al. (2022) conducted a study to explore the anticancer effects of scabertopin on three bladder cancer cell lines (J82, T24, RT4, and 5637). The results showed that scabertopin treatment significantly suppressed the proliferation of all three bladder cancer cell lines. Further mechanistic analyses on the J82 cell line revealed that scabertopin induced apoptotic cell death in this cell line but no marker of apoptosis, including Bax, Bcl-2, and caspases 3, 8, and 9, were changed in that study. They also reported that scabertopin-treated J82 cells did not die by apoptosis, ferroptosis, or pyroptosis.

### 4.24 Sesquiterpene lactone 3

In a study by Zhang X. W. et al. (2018), sesquiterpene lactone 3 (SL3), as a bioactive component *Artemisia argyi,* was shown to have anticancer effects on two gastric carcinoma cell lines AGS and MGC803. Further mechanistic study demonstrated that SL3 induced apoptosis in both cancer cell lines by activating cleavage of caspase 3 and PARP enzymes. However, no other apoptosis marker was evaluated.

### 4.25 Uvedafolin

Uvedafolin is a sesquiterpene lactone isolated from the leaves of *Smallanthus sonchifolius*. Uvedafolin has been claimed to induce apoptotic cell death in cervical cancer HeLa cells. Uvedafolin treatment of HeLa cells has led to cell cycle arrest at the G2/M phase and induced apoptosis by a mitochondrial-related mechanism that activates caspase 3, 7, and 9, dissipates MMP, and increases cytochrome C release into the cytosol (Kitai et al., 2017).

### 4.26 Vernolactone

Vernolactone is a sesquiterpene lactone compound found in *Vernonia zeylanica*. Mendis et al. (2019) isolated this sesquiterpene from chloroform and ethyl acetate fractions of *V. zeylanica* and explored its anti-cancer effects on three breast cancer cell lines (MCF -7, MDA-MB-231, SKBR-3). The results showed that vernolactone was an anti-proliferative agent against SKBR-3 and MDA-MB-231 breast cancer cells, with minimal effect on MCF-7 and normal breast epithelial cell line MCF-10A. Further analyses evidenced apoptotic cell death indexes such as morphological alterations, DNA fragmentation, increased activity of caspases 3 and 7, upregulation of Bax, and downregulation of survivin. In a second study, the effects of vernolactone on apoptosis and autophagy induction in human embryonic carcinoma stem-like cells (NTERA-2) were examined. The results suggested that vernolactone has the ability to induce both autophagy and apoptosis, which was affirmed by a significant increase in the activities of caspase 3 and caspase 7 and a significant decrease in the level of survivin in NTERA-2 cells at the concentrations of 2 μg/mL and 4 μg/mL (Abeysinghe et al., 2019).

## 5 Concluding remarks

Most of the studies available in the literature have evaluated the effect of sesquiterpene lactones on the mitochondrial or intrinsic pathway of apoptosis. It is not clear why the researchers have assessed the intrinsic pathway of apoptosis in their research projects. Only 16 studies were found that have examined both pathways of apoptosis. As indicated in the literature, therapy resistance is a major challenge during cancer therapy approaches (Gupta et al., 2022). This resistance may arise from the cross-talks between different signaling pathways in the intracellular spaces (Yamaguchi et al., 2014; Sun et al., 2016). Cancer cells sometimes escape therapeutic agents by switching from one pathway to another (Noubissi et al., 2018). Thus, the drugs with more than two targets in the cancer cells are more competent to cease cancer growth and progression. This present review article also highlighted that only 27 studies have investigated the anti-cancer effect of sesquiterpene lactones on an animal model. Because of many ethical and practical concerns related to human studies, animal models have been essential in cancer research. Although the successful translation of the outcomes from animal studies to clinical cancer trials is about 8%, animal models could be considered a good way to obtain essential information from the *in-vivo* effects of cancer therapeutics (Mak et al., 2014).

## 6 Future directions

Future studies on pro-apoptotic effects of natural or chemical agents need to assess at least key factors of both intrinsic and extrinsic pathways such as Fas receptors, Bid, and caspases 9, 8, and 3. In addition, the expression levels of c-FLIP and IAPs are of great importance in the investigation of the apoptosis-inducing activity of a drug candidate. Also, future studies on anticancer activities of sesquiterpene lactones should evaluate these activities in an established model of the disease to affirm *in-vitro* data. Combination therapy is a therapeutic strategy that combines two or more therapeutic compounds to enhance the efficacy of each drug in treating a single disease (Correia et al., 2021). This approach is more appropriate for anti-cancer drugs because it may lead to targeting several pathways synergistically and could potentially reduce drug resistance (Bayat Mokhtari et al., 2017). Thus, it appears that combination therapy using different phytochemicals or phytochemicals and chemical compounds is a promising strategy to overcome therapy resistance in cancer therapy. Bioavailability is one of the major drawbacks in therapeutic agents with natural origin (Estrela et al., 2017). Therefore, it is necessary to conduct pharmacokinetic studies to assess the bioavailability of therapeutic phytochemicals to select the best one for further therapy evaluation. One of the promising and effective approaches for increasing the bioavailability and targetability of drugs, especially natural agents, is the use of nanomaterial structures (Salehi et al., 2020; Solanki et al., 2022).

## 7 Clinical gaps

Although sesquiterpene lactones have been identified as a potential anti-cancer agent based on pre-clinical data, they may not be utilized as a first-line therapy for cancer patients in clinical settings due to some drawbacks. One of the limitations comes from the lack of preclinical pharmacological studies to determine the different aspects of the pharmacokinetics of this bioactive compound. Another drawback may arise from the limited number of *in vivo* studies and translational investigations with this compound to affirm effective doses in human subjects. As mentioned before, bioavailability is a big challenge in the use of natural compounds. One potential solution for this limitation could be the use of nano pharmaceutical approaches to incorporate sesquiterpene lactones into specific and well-characterized nanoparticles to enhance the bioavailability of the drug and appropriate delivery to the target organs. The other obstacle to using these therapeutic agents is the lack of clinical studies that establish the efficacy, adverse effects, and toxic impacts of these compounds on human subjects. Although sesquiterpene lactones are not good enough to be used as first-line in cancer therapy, they may be appropriate to be utilized as add-on therapy in combination with chemotherapeutic compounds. However, it is necessary to evaluate the synergistic or antagonistic interactions between sesquiterpene lactones with chemotherapeutics.
